# An African *Salmonella* Typhimurium ST313 sublineage with extensive drug-resistance and signatures of host adaptation

**DOI:** 10.1038/s41467-019-11844-z

**Published:** 2019-09-19

**Authors:** Sandra Van Puyvelde, Derek Pickard, Koen Vandelannoote, Eva Heinz, Barbara Barbé, Tessa de Block, Simon Clare, Eve L. Coomber, Katherine Harcourt, Sushmita Sridhar, Emily A. Lees, Nicole E. Wheeler, Elizabeth J. Klemm, Laura Kuijpers, Lisette Mbuyi Kalonji, Marie-France Phoba, Dadi Falay, Dauly Ngbonda, Octavie Lunguya, Jan Jacobs, Gordon Dougan, Stijn Deborggraeve

**Affiliations:** 1 Department of Biomedical Sciences, Institute of Tropical Medicine, Nationalestraat 155, 2000 Antwerp, Belgium; 2 Wellcome Sanger Institute, Wellcome Genome Campus, Hinxton, Cambridge, CB10 1SA UK; 3 Laboratory of Medical Microbiology, Vaccine & Infectious Disease Institute, University of Antwerp, Antwerp, Belgium; 4 Department of Medicine, Addenbrooke’s Hospital, University of Cambridge, Cambridge, CB2 0SP UK; 5 Department of Vector Biology, Liverpool School of Tropical Medicine, Pembroke Place, Liverpool, L3 5QA UK; 6 Department of Clinical Sciences, Institute of Tropical Medicine, Nationalestraat 155, 2000 Antwerp, Belgium; 7 Centre for Genomic Pathogen Surveillance, Wellcome Sanger Institute, Wellcome Genome Campus, Hinxton, Cambridge, CB10 1SA UK; 8 Department of Microbiology and Immunology, KU Leuven, Herestraat 49—box 1030, 3000 Leuven, Belgium; 9 Department of Microbiology, National Institute for Biomedical Research, Av. De La Démocratie no, 5345 Kinshasa, Democratic Republic of the Congo; 10 Department of Microbiology, University Hospital of Kinshasa, Kinshasa, Democratic Republic of the Congo; 11 Department of Pediatrics, University Hospital of Kisangani, Avenue Munyororo C/Makiso, Kisangani, BP 2012 Democratic Republic of the Congo

**Keywords:** Phylogeny, Antimicrobial resistance, Bacterial genetics, Bacterial infection, Developing world

## Abstract

Bloodstream infections by *Salmonella enterica* serovar Typhimurium constitute a major health burden in sub-Saharan Africa (SSA). These invasive non-typhoidal (iNTS) infections are dominated by isolates of the antibiotic resistance-associated sequence type (ST) 313. Here, we report emergence of ST313 sublineage II.1 in the Democratic Republic of the Congo. Sublineage II.1 exhibits extensive drug resistance, involving a combination of multidrug resistance, extended spectrum β-lactamase production and azithromycin resistance. ST313 lineage II.1 isolates harbour an IncHI2 plasmid we name pSTm-ST313-II.1, with one isolate also exhibiting decreased ciprofloxacin susceptibility. Whole genome sequencing reveals that ST313 II.1 isolates have accumulated genetic signatures potentially associated with altered pathogenicity and host adaptation, related to changes observed in biofilm formation and metabolic capacity. Sublineage II.1 emerged at the beginning of the 21st century and is involved in on-going outbreaks. Our data provide evidence of further evolution within the ST313 clade associated with iNTS in SSA.

## Abstract (web summary)

Invasive non-typhoidal *Salmonella* (iNTS) infections are dominated by antibiotic resistant isolates of the sequence type (ST) 313. Here, the authors identify the ST313 sublineage II.1 in the Democratic Republic of the Congo exhibiting extensive drug resistance and genetic signatures potentially associated with host adaptation.

## Introduction

S*almonella enterica* subspecies *enterica* serovar Typhimurium (*S*. Typhimurium) and other non-typhoidal *Salmonella* are common causes of gastrointestinal infections in people living in industrialized countries. However, in sub-Saharan Africa (SSA), invasive non-typhoidal *Salmonella* (iNTS) bloodstream infections are common^1,2^ totalling ~3.4 million cases annually, with *S*. Typhimurium being responsible for approximately two-thirds of these cases. The fatality rate in iNTS can be extremely high^3^.

In SSA, iNTS patients often do not suffer from diarrhoea but instead display symptoms of fever and septicaemia^4^. There has been no proven zoonotic source of ST313 infections, and human to human transmission has been postulated^5,6^. The disease disproportionately affects children under 5-years old and human immunodeficiency virus (HIV) positive adults^7^.

Whereas the majority of *S*. Typhimurium associated with gastroenteritis in developed countries belong to sequence types (ST) 19 and 34, *S*. Typhimurium iNTS in SSA are predominantly of ST313^8^. The population structure of *S*. Typhimurium ST313 is dominated by two clonal lineages, named I and II, that sequentially spread across SSA over the past 40 years. The success of these ongoing pandemics has been attributed to resistance to antibiotics and the emergence of HIV^9^. The majority of iNTS *S*. Typhimurium isolated in the past 10 years have been of ST313 lineage II^10–12^.

*S*. Typhimurium ST313 isolates are predominantly multidrug resistant (MDR), implying co-resistance to the three former first line antibiotics ampicillin, trimethoprim/sulfamethoxazole and chloramphenicol^13,14^. Two recent genomics-based studies reported the acquisition of extended-spectrum β-lactamases (ESBLs) conferring resistance to the third generation cephalosporin ceftriaxone among *S*. Typhimurium ST313 lineage II isolates from Malawi and Kenya^10,11^. Ceftriaxone is a recommended antibiotic to treat complicated iNTS, while the fluoroquinolone ciprofloxacin is recommended for uncomplicated iNTS^15^.

The genomes of *S*. Typhimurium ST313 show evidence of specialization towards a narrow host range by pseudogenization in a pattern that resembles that found in the host restricted typhoidal *Salmonella enterica* subspecies *enterica* serovar Typhi (*S*. Typhi)^4,16,17^. The genotypic differences between ST313 and ST19 isolates have been confirmed as phenotypes associated with host virulence and other traits, including biofilm formation which has been postulated to influence both survival in the environment and macrophages^9,18–22^.

Here, we report the emergence of an extensively drug resistant (XDR) ST313 sublineage we name II.1, which is currently causing bloodstream infections in the Democratic Republic of the Congo (DRC) and represents >10 % of all *S*. Typhimurium isolated in the Kongo Central Province^14^. This sublineage is associated with a combination of MDR, ESBL production and resistance to azithromycin (AZI). Additionally, whole-genome sequencing of multiple genomes identifies signatures associated with pathogenicity, metabolism and potentially host adaptation.

## Results

### An XDR *S*. Typhimurium ST313 sublineage II.1 is emerging

**Fig. 1:**
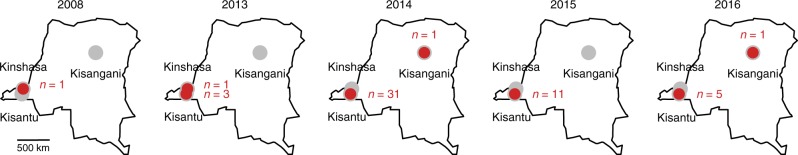
Geographical origin of *S*. Typhimurium showing azithromycin resistance in the Democratic Republic of the Congo (DRC). Red dots with numbers (*n*) of the included resistant strains in the respective surveillance sites (Kinshasa, Kisangani and Kisantu). The map of DRC was constructed using the maps package in R^80^

Invasive *S*. Typhimurium have been isolated in various hospital sites across DRC during ongoing microbial surveillance of bacterial bloodstream infections^14,23,24^. As third-generation cephalosporins and AZI have been used in treatment, we have monitored susceptibility using both phenotypic and genetic approaches. Consequently, to investigate the emerging phenotype of ESBL production and AZI resistance, a selection of 81 *S*. Typhimurium isolates from the region were subjected to whole-genome sequence analysis. Their year and place of origin, the age of the patient and their phenotypic antimicrobial susceptibility are listed in Supplementary Data 1. Of this panel, 54 *S*. Typhimurium isolates exhibited AZI resistance and were ESBL positive; all except three were also MDR and are thus classifiable as XDR. The isolates were collected from 2008 to 2016 in western (Kisantu, Kongo Central Province *n* = 50; Kinshasa *n* = 2) and north-eastern DRC (Kisangani, Tshopo Province: *n* = 2) (Fig. 1). Minimum inhibitory concentration (MIC) values for AZI resistance were between 32 and >256 mg L^−1^. Two of these isolates, 5390_4 (Kisangani, 2016) and 2735 (Kinshasa, 2008), showed decreased susceptibility to the fluoroquinolone ciprofloxacin (decreased ciprofloxacin susceptibility (DCS), MIC values of 0.38 and 0.19 mg L^−1^against ciprofloxacin, respectively). These isolates also exhibited resistance to the quinolone pefloxacin, and susceptibility (5390_4) or resistance (2735) to the quinolone nalidixic acid. The other 27 *S*. Typhimurium isolates form a representative local context, originating from bloodstream infections in the same surveillance sites in DRC (see Supplementary Note 1). They were isolated between 2007 and 2016, and showed no ESBL production nor resistance to AZI. The majority of these isolates (22 out of 27) were MDR. One isolate, 16755_3 (Kisantu, 2016), exhibited DCS with a MIC value of 0.19 mg L^−1^.

Multilocus sequence typing (MLST) confirmed that all belong to ST313. To provide context for the genomic analysis, 153 African^9,11,12,25,26^ and 42 non-African^9^ *S*. Typhimurium genomes were included in the overall analysis. The complete list of 276 analysed *S*. Typhimurium genomes with the year, place and source of isolation is presented in Supplementary Data 2.

**Fig. 2:**
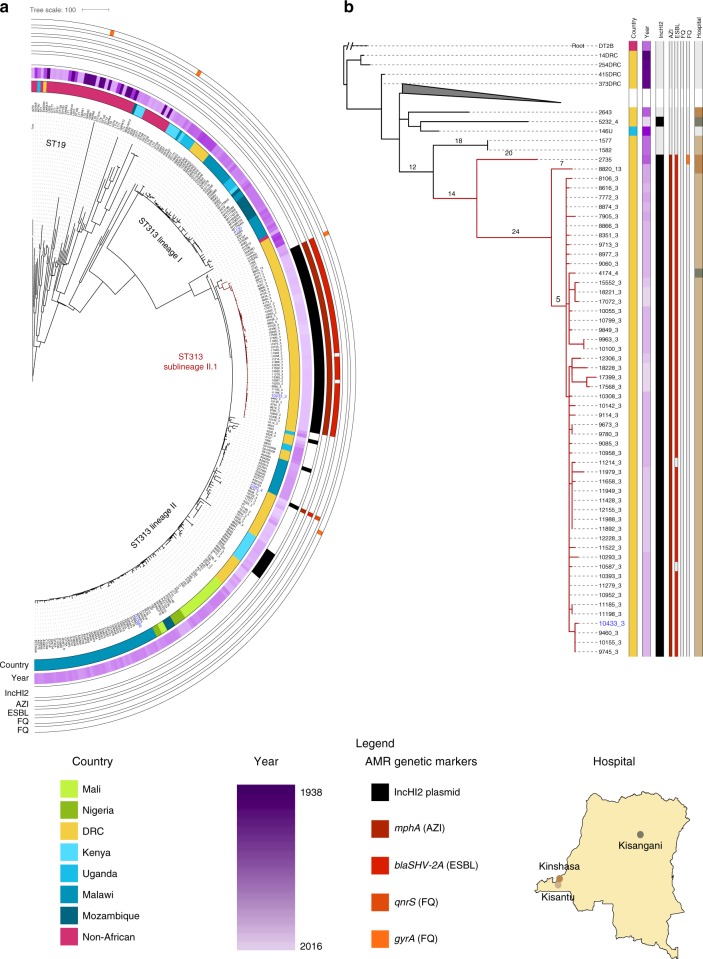
The population structure of *S*. Typhimurium ST313 with emergence of sublineage II.1. **a** Maximum likelihood phylogenetic tree based on the 81 genome sequences from this study and 153 African and 42 non-African publicly available *S*. Typhimurium strains (summarized in Supplementary Data 2). Sequencing reads were mapped to *S*. Typhimurium ST313 lineage II reference strain D23580^56^. The tree is based on 62,884 chromosomal SNPs. Branches of *S*. Typhimurium ST313 sublineage II.1 are coloured in red. Metadata is visualized on the concentric rings in compliance to the legend, from the inside to outside; 1. Country of origin, 2. Year of isolation, 3. Presence of IncHI2 replicon, 4–8. Presence of multidrug resistance markers (MDR; *bla, cat, sul, dfrA*), *mphA, blaSHV-2A, qnrS* and *gyrA* antimicrobial resistance markers (AMR). Reference strains A130 (lineage I), D23580 (lineage II) and 10433_3 (sublineage II.1), as well as strain 5390_4, are indicated in blue. Branch lengths represent the number of SNPs as indicated in the scale bar. The tree is publicly available on MicroReact (https://microreact.org/project/xS5Xw6b3A). **b** Maximum likelihood phylogenetic tree of all *S*. Typhimurium ST313 lineage II strains included in this study, based on mapping to sublineage II.1 reference strain 10433_3 (this study). The tree is based on 1207 chromosomal SNPs. A collapsed branch is annotated with a grey triangle. The tree is rooted with *S*. Typhimurium strain DT2B, a European ST313 strain. Branches of *S*. Typhimurium ST313 sublineage II.1 are coloured in red. Metadata is visualized in lanes in compliance to the legend, from left to right; 1. Country of origin, 2. Year of isolation, 3. Presence of IncHI2 replicon, 4–8. Presence of multidrug resistance markers (MDR; *bla, cat, sul, dfrA*), *mphA, blaSHV-2A, qnrS* and *gyrA* AMR markers, 9. Location in the Democratic Republic of the Congo (DRC). Reference strain 10433_3 (sublineage II.1) is indicated in blue. Branch lengths are indicated and represent the number of SNPs. The map of DRC was constructed using the maps package in R^80^

This analysis revealed a sublineage II.1 in DRC defined by the most recent common ancestor of the monophyletic sublineage of 51 XDR isolates (Fig. 2a). One isolate, 5390_4, originating from Kisangani (2016) while being MDR, ESBL producing, AZI resistant and showing DCS, falls outside sublineage II.1, but is part of the clonal lineage II. All 27 control isolates also fall into lineage II, with two older isolates from Kisantu (2009) being most closely related to sublineage II.1 (1577 and 1582).

The *S*. Typhimurium ST313 lineage I and II in our study accumulated 225 and 216 conserved single nucleotide polymorphisms (SNPs) respectively in their core genomes compared to their most recent common ancestor (Supplementary Fig. 1), which is comparable to previous observations^17^. In comparison to the lineage II clade, all sublineage II.1 isolates, except 2735, accumulated an additional 38 conserved core SNPs, and show a clonal structure with little substructure (Fig. 2b). Of note, the two isolates showing an AZI MIC value >256 mg L^−1^ form a monophyletic clade within sublineage II.1 (17399_3 and 17568_3). No SNPs were acquired in known genes involved in macrolide resistance^27^, but both isolates have a G118V amino acid substitution in the ABC-transporter protein BtuC. This SNP, in addition to the presence of *mphA*, might be involved in the observed increased AZI resistance^28^. Cephalosporin resistant ST313 lineage II infections were previously reported from Kenya and Malawi^10–12^. The XDR isolates we describe here form ST313 sublineage II.1.

**Fig. 3:**
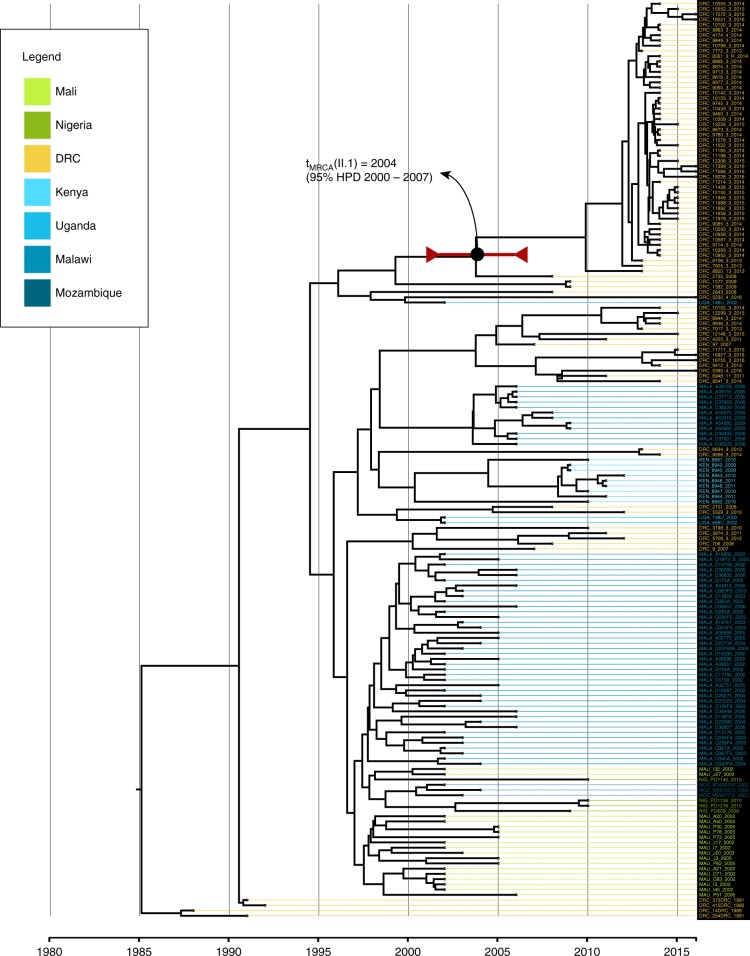
Bayesian time-tree of *S*. Typhimurium ST313 lineage II Bayesian maximum clade credibility phylogeny of African *S*. Typhimurium ST313 lineage II and sublineage II.1 isolates. The time-tree is based on 1187 SNP differences detected across the core genome of 175 lineage II and II.1 sequenced isolates. The tree was visualized and coloured in Figtree v1.4.2, with the horizontal axis representing the years. A divergence date (median estimate and its respective 95% HPD) is indicated for the ST313 II.1 sublineage. Tree tips are colour coded according to their country of origin (coloured by legend at top)

The putative origin of the ST313 lineage II was previously predicted to be the DRC^9^. Consequently, we applied a temporal reconstruction using BEAST2 to the *S*. Typhimurium ST313 lineage II and II.1 isolates. This limited analysis indicates that sublineage II.1 may have emerged in the DRC around 2004 (95% highest probability density (HPD) interval: 2000–2007) (Fig. 3).

### Lineage II.1 harbours an IncHI2 resistance plasmid, pSTm-ST313-II.1

A comprehensive resistome, composed of all catalogued genetic determinants for antimicrobial resistance (AMR), was bioinformatically extracted from the sequencing data (Supplementary Data 2). This analysis indicates that the MDR phenotype observed in *S*. Typhimurium ST313 lineage II and sublineage II.1 isolates is associated with genes predicted to confer resistance to chloramphenicol (*catA*), ampicillin (*blaTEM1*) and trimethoprim (*dfrA*). While lineage II is linked to the presence of *dfrA1*, this allele is replaced by *dfrA14* in sublineage II.1. XDR sublineage II.1 isolates harbour *blaSHV-2A* and *mphA* genes, respectively potentially associated with resistance to cephalosporins and azithromycin, respectively. Identical *blaSHV-2A* and *mphA* genes are present in the ESBL positive, AZI resistant lineage II isolate from Kisangani (5390_4). One sublineage II.1 isolate, 2735, showed DCS (MIC value ciprofloxacin = 0.19 g L^−1^) in addition to AZI resistance and ESBL production and harbours a mutation in *gyrA* (D87N) associated with DCS. DCS combined with ESBL positivity and AZI resistance was also observed in the lineage II isolate 5390_4 (Kisangani, 2016, MIC value ciprofloxacin = 0.38 g L^−1^), which has acquired a *qnrS* gene. The lineage II isolate 16755_3 (Kisantu, 2016, MIC value ciprofloxacin = 0.19 g L^−1^) showed DCS but no ESBL activity nor AZI resistance and harbours a S83Y mutation in *gyrA*.

**Fig. 4:**
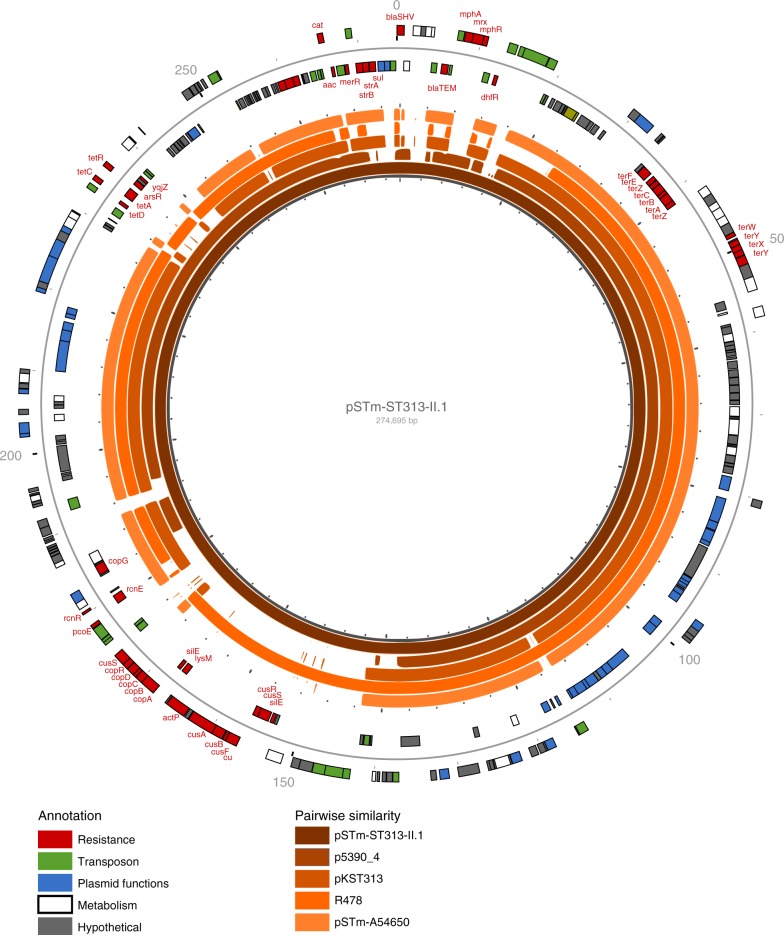
IncHI2 resistance plasmid pSTm-ST313-II.1. The genetic makeup of the pSTm-ST313-II.1 resistance plasmid from isolate 10433_3 is given. pSTm-ST313-II.1 is 274,695 nucleotides long and has 290 annotated genes. Annotations are shown on the outer circle and coloured by gene function: resistance genes (red), plasmid functions (blue), transposon related genes (green), metabolism functions (white) and hypothetical genes (grey). The inner five circles show pairwise similarity regions of 100% with previously reported IncHI2 plasmids in *S*. Typhimurium ST313 (pKST313 from Kenya (LN794248) and pSTm-A54650 from Malawi (LK056646)), a draft assembly from the IncHI2 plasmid of strain 5390_4 from Kisangani (this study) and R478 IncHI2 from *Serratia marcescens* isolated in the USA (BX664015)

All sublineage II.1 isolates as well as the lineage II 5390_4 isolate (ESBL + AZI) from Kisangani harbour an IncHI2 plasmid. IncHI2 plasmids have been reported previously in other *S*. Typhimurium ST313 isolates from Kenya (pKST313) and Malawi (pSTm-A54650)^10,11^. We have named the ST313 sublineage II.1 IncHI2 plasmid pSTm-ST313-II.1. The full pSTm-ST313-II.1 plasmid sequence associated with sublineage II.1 was determined using PacBio sequencing of isolate 10433_3 (Kisantu, 2014). pSTm-ST313-II.1 is 274,695 nucleotides (Fig. 4) and is highly conserved among all sublineage II.1 isolates, differing by ~5 single SNPs between isolates. The known AMR determinants of ST313 sublineage II.1 are located on pSTm-ST313-II.1, alongside genes associated with heavy metal resistance against silver and copper. pSTm-ST313-II.1 encodes a potentially active TraB conjugation protein and conjugation operon^29^.

pSTm-ST313-II.1 exhibits significant similarity to R478 (99% identity over 86% coverage of pSTm-ST313-II.1), a self-transferable IncHI2 plasmid isolated from *Serratia marcescens* in 1969 in the USA^30^. Pairwise sequence comparisons of pSTm-ST313-II.1 with the other IncHI2 plasmids (1) pKST313 from Kenya, (2) pSTm-A54650 from Malawi, a (3) draft assembly from the IncHI2 plasmid of 5390_4 from Kisangani, and R478 revealed the significant similarity between the plasmid backbones (Fig. 4 and Supplementary Table 1). The less conserved regions of pSTm-ST313-II.1 include AMR genes, flanked by transposon-associated regions (Fig. 4).

### Sublineage II.1 exhibits signatures of host adaptation

**Fig. 5:**
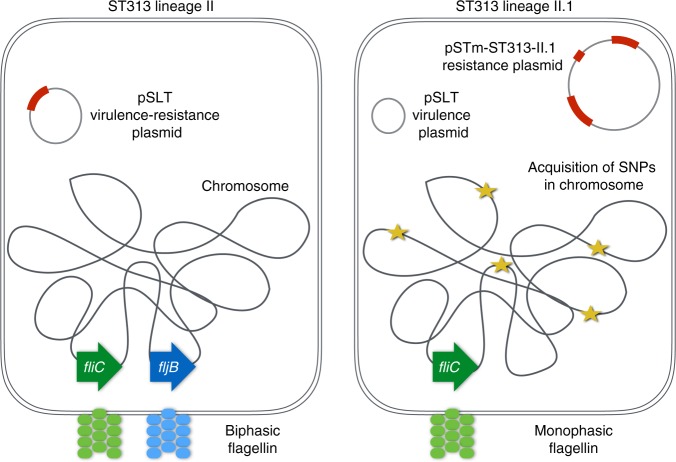
Genomic changes in *S*. Typhimurium ST313 sublineage II.1 versus ST313 lineage II. A schematic overview of the genomic differences between *S*. Typhimurium ST313 sublineage II.1 versus ST313 lineage II is shown. The genetic changes in *S*. Typhimurium sublineage II.1 include the acquisition of a resistance plasmid, pSTm-ST313-II.1, the loss of the flagellin *fljB* gene and the acquisition of single nucleotide polymorphisms (SNPs) in the chromosome sequence

ST313 sublineage II.1 harbours multiple chromosomal sequence differences in comparison to ST313 lineage II isolates (Fig. 5). A deletion of 1076 nt was observed in the chromosome of ST313 sublineage II.1, resulting in loss of *fljB*. The *fljB* gene codes for the phase 2 flagellin protein which polymerizes to form the bacterial flagella (Supplementary Fig. 2). Loss of *fljB* (FljB) was confirmed at the DNA and protein levels (Supplementary Fig. 3). Consequently, in contrast to their biphasic ancestors, sublineage II.1 isolates are monophasic as they only harbour the phase 1 flagellin gene, *fliC*.

We annotated the SNPs that were acquired in ST313 sublineage II.1 relative to lineage II (Supplementary Tables 2–4). Of these SNPs, 19 are present in coding regions and cause predicted non-synonymous mutations in the protein sequence (Supplementary Tables 2–4). Seven of these 19 SNPs are located in conserved Pfam protein domains resulting in deviant bitscores. Consequently, we prioritized these SNPs as the most likely to cause functional defects. The affected genes are geranyltranstransferase *ispA*, methyl-accepting chemotaxis protein *trg_1*, precorrin-3B C17-methyltransferase *cbiH*, propanediol dehydratase reactivation protein, outer membrane assembly protein *asmA*, putative multidrug export ATP-binding/permease protein SAV1866 and putative diguanylate cyclase *yhjK*. These genes have been linked to host-adaptation (*cbiH*)^16^, virulence-associated processes (*asmA*)^31^, and chemotaxis during infection (*trg_1*)^32^. Of note, these molecular processes are also linked to pseudogene accumulation in the phylogeny from *S*. Typhimurium ST19 to ST313 lineage II^17^.

**Fig. 6:**
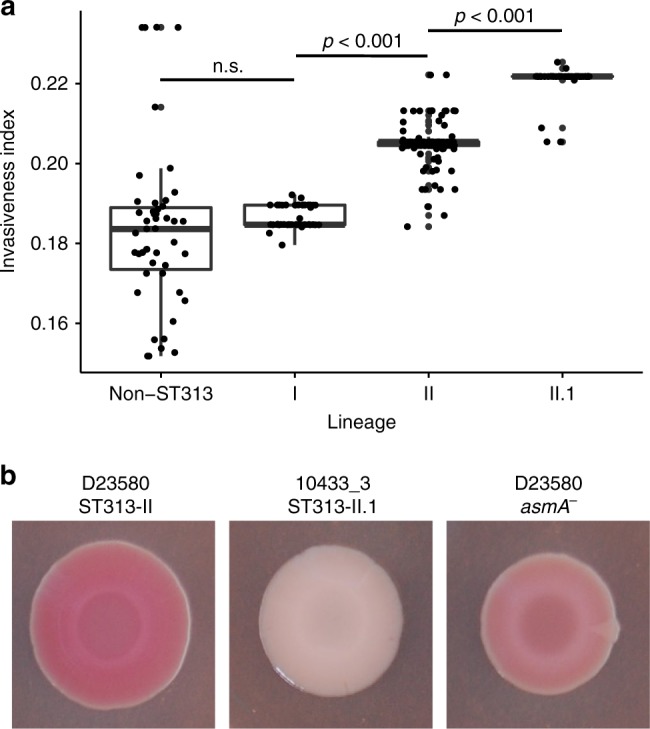
Signatures for host adaptation of *S*. Typhimurium ST313 Sublineage II.1. **a** Invasiveness index values for all *S*. Typhimurium sequences included in this study, grouped into lineage, as calculated by the method of Wheeler et al.^33^. Summary of statistics on different clades: Non-ST313: median = 0.184, standard deviation (SD) = 0.093; Lineage I: median = 0.185, SD = 0.003; Lineage II: median = 0.205, SD = 0.005; Lineage II.1: median = 0.222, SD = 0.004. The groups were compared using a Mann Whitney *U-*test. Boxplot centre lines represent median values; box limits represent upper and lower quartiles; whiskers represent the 1.5 interquartile range and points represent the outliers. Source data are provided as a Source Data file. **b** Red, dry and rough (rdar) morphotype^75^ of *S*. Typhimurium ST313 lineage II strain D23580^56^, *S*. Typhimurium ST313 lineage II.1 strain 10433_3 (this study) and a *S*. Typhimurium ST313 lineage II D23580 *asmA* knock-out strain (this study). Source data are provided as a Source Data file

As our interpretations are likely biased by the available literature, we applied a data mining approach to obtain a more objective evaluation of the association between the acquired SNPs in sublineage II.1 and invasiveness. Hereto, the *Salmonella* invasiveness index of each strain was calculated, which is based on the genomic signatures for *S. enterica* associated with adaptation to an invasive lifestyle^33^. We observe an increase in invasiveness index from non-ST313 and ST313 lineage I isolates to lineage II, and a significant further increase of the invasiveness index from lineage II to lineage II.1 (Fig. 6a, Supplementary Fig. 3).

In addition to the observed genomic differences, *S*. Typhimurium ST313 sublineage II.1 isolates exhibited measurably different phenotypes compared to *S*. Typhimurium ST313 lineage II. A red dry and rough (rdar) biofilm assay was performed that uses agar plates with stains that reveal extracellular matrix production in colonies. In this assay, *S*. Typhimurium ST313 lineage II isolates lost the rough morphotype compared to ST19, changing from a rdar to a brown and smooth (bas) phenotype. This phenotype was shown previously to be due to defective production of cellulose, one of the two major extracellular components of *Salmonella* biofilms^18,20^. Interestingly, *S*. Typhimurium ST313 lineage II.1 show a further defect in biofilm formation. Sublineage II.1 isolates have a smooth and white (saw) colony morphotype in this assay, whereas sublineage II isolates have a brown and smooth (bas) morphotype (Fig. 6b). This phenotypic defect is consistent across all isolates from sublineage II.1 and resembles the *S*. Typhi morphotype. The exception was isolate 2735 (Kinshasa, 2008), the phylogenetically oldest representative of sublineage II.1 (Supplementary Fig. 4)^19^. Among the SNPs that were acquired in sublineage II.1 compared to lineage II, none were located in or near genes responsible for the production of curli, which is another major biofilm compound. Mutagenesis experiments showed that the introduction of a null mutation into the *asmA* gene of *S*. Typhimurium ST313 lineage II D23580 partly recreated the sublineage II.1 biofilm phenotype (Fig. 6b), suggesting that the non-synonymous SNP in *asmA* might contribute to the biofilm defect. *asmA* encodes an outer membrane protein that was previously described to be involved in the invasion of epithelial cells^31^. Targeted mutations in *wzxC*, *yhjJ* and *yhjK* in D23580 had no obvious effect on the biofilm phenotype (Supplementary Fig. 5).

While *Salmonella* causing gastrointestinal infections typically have a relatively large metabolic capacity, strains causing invasive infections generally have a more limited capacity^34^. We used Biolog Phenotype Microarrays to assess growth on 192 different metabolic compounds between two representative isolates of both lineage II and lineage II.1. Lineage II.1 isolates showed significant lower metabolic capacity for carbon compounds compared to lineage II isolates (Supplementary Data 3). The effect was most pronounced for D-galactonic acid γ-lacton (Supplementary Fig. 6).

We also assessed the phenotypic behaviour of five representative sublineage II.1 isolates in in vitro and in vivo models. Human macrophage infections and intravenous and oral mouse infections did not show significant differences between the lineages, although two sublineage II.1 isolates showed an overall lower infection of THP macrophages and cell counts in mouse deep tissue after intravenous infections (Supplementary Figs. 7–9).

## Discussion

Here, we report the emergence of *S*. Typhimurium ST313 sublineage II.1 from the DRC showing XDR. The World Health Organization (WHO) has listed *Salmonella* spp. as one of the pathogens for which new antibiotics are urgently needed^35^. In addition to the MDR phenotype of lineage II^9^, sublineage II.1 is associated with ESBL activity, AZI resistance and occasional DCS, thereby giving rise to the first lineage of XDR iNTS. As there is no XDR definition for iNTS yet, we extrapolate the definition from XDR *S*. Typhi, recently defined as resistant to five antibiotics (observed as MDR combined with resistance against fluoroquinolones and ESBL activity), thereby using the same nomenclature as for other bacterial pathogens^36,37^. The XDR iNTS isolates from this study show MDR in combination with resistance to the two alternative treatment options ceftriaxone and AZI.

Sublineage II.1 was identified in three independent surveillance sites in the DRC, suggesting this sublineage is relatively widespread in the region, although further surveillance is required to confirm the burden^14,38–40^.

All except three of the *S*. Typhimurium ST313 lineage II.1 isolates described here fall into the XDR category defined for *S*. Typhi^36,37^. Within the DRC setting, the only available antibiotics for treatment of XDR iNTS infections are fluoroquinolones. Importantly, DCS iNTS is present in DRC, with one pan-resistant (XDR + DCS) isolate identified in this study. The emergence of this XDR iNTS sublineage II.1 increases the urgency for advancements in other strategies, such as vaccination, to combat the disease.

Antibiotic use might have provided the selection pressure driving the emergence of this sublineage. Within SSA, the antibiotic AZI has been used in mass drug administrations for the elimination of Trachoma, and has also recently been tested to reduce childhood mortality^41^. In the DRC, it is not clear which antibiotics are in routine use, as only 30% of patients have access to the regular healthcare system, and 40% rely on self-medication^42^. MDR in ST313 sublineage II.1 is linked here with the IncHI2 plasmid pSTm-ST313-II.1. Intriguingly, previous reports have associated iNTS *S*. Typhimurium in SSA with IncHI2 plasmids^10,11,43^.

The acquisition of antibiotic resistance and the accumulation of genomic signatures associated with host adaptation by sublineage II.1 suggest further specialization to a human niche^16,17^. Sublineage II.1 are monophasic, a phenotype similar to typhoidal *Salmonella*^44^. A large recombination event took place at the *fljB* locus, resulting in loss of phase II flagellin production, while the phase I flagellin gene *fliC* remained intact. Bacterial flagellin plays an important role in the host innate immune response and is a stimulator of innate immunity through Tlr5^45,46^. Strikingly, a pandemic of monophasic *S*. Typhimurium ST34 causing gastrointestinal infections has recently been identified, with some strains of this pandemic causing bloodstream infections in Vietnam^47,48^. Sublineage II.1 have a reduced capacity to form multicellular communities compared to lineage II^18^, again with similarity to *S*. Typhi^19^. Bacterial biofilms are important for both resistance to environmental stresses and survival outside the host. *S*. Typhimurium ST313 sublineage II.1 isolates also show a lower capacity for the metabolism of carbon sources, in line with the findings that *S*. Typhimurium ST313 has a reduced metabolic capacity compared to *S*. Typhimurium ST19^17^.

Survival in macrophages was experimentally assessed, given the observed mutations in sublineage II.1, such as loss of *fljB*, which can affect macrophage survival^20^. However, we did not find significant differences between lineage II and sublineage II.1 in macrophage infection assays. Likewise, II.1 isolates did not show a reproducible increased colonization of deeper tissue during intravenous or oral mouse infections. Although our genomic, biofilm and metabolic data show signatures for increased human adaption of lineage II, *S*. Typhimurium ST313 lineage II.1 isolates are thus far not host restricted.

In conclusion, in this study we identified an on-going outbreak of sublineage II.1 emerging from the current African *S*. Typhimurium ST313 pandemic. Sublineage II.1 is associated with XDR driven by an IncHI2 plasmid named pSTm-ST313-II.1, harbouring resistance to AZI and ESBL production. In addition, sublineage II.1 also shows evolutionary signatures associated with host adaptation. Extended bloodstream surveillance in the endemic regions of *S*. Typhimurium ST313 will be crucial to further track the spread of XDR ST313 sublineage II.1 and to timely detect the emergence of sublineages with novel antibiotic resistance profiles.

## Methods

### Isolate selection from bloodstream surveillance

The DRC isolates originated from blood cultures sampled at the referral hospital of Saint Luc in Kisantu (Kongo Central province), the University Hospital of Kinshasa, the referral Hospital St. Joseph and Monkole Hospital from Kinshasa (Kinshasa province), the referral hospital of Bwamanda (Tshopo province), the referral hospital of Kabondo and the university hospital of Kisangani (CUKIS) and associated health centres (Sud-Ubangi province) in the DRC^14,24,40^. These hospitals have participated in the microbiological surveillance network since 2007 coordinated by the National Institute of Biomedical Research (INRB) in Kinshasa, DRC, in collaboration with the Institute of Tropical Medicine (ITM) in Antwerp, Belgium. Blood cultures were sampled in patients suspected of bloodstream infections according to standard indications and methods as described elsewhere^14^.

All available AZI resistant *S*. Typhimurium available to this study were included (*n* = 54). A sample of 27 representative non-AZI resistant *S*. Typhimurium isolates were selected as controls for this analysis. Most health facilities across the country lack capacity for diagnosing bacterial bloodstream infections and we have therefore no information about bloodstream infections elsewhere in DRC except as part of outbreak research^38,39^. The surveillance sites included in this study were not consistently active because of stock ruptures, staff movements, funding and insecurity. All isolates were stored in tubes of Trypticase Soya Agar (Oxoid, Basingstoke, UK) and shipped to ITM for confirmation and further identification.

At ITM, isolates biochemically confirmed as *Salmonella* spp. were serotyped using commercial antisera (Sifin, Berlin, Germany) according to the Kauffmann-White scheme^49^. A representative selection of the isolates (10%) was sent to the National Reference Centre in Belgium for confirmation of serotype. Antibiotic susceptibility testing of all isolates was done by disk diffusion (Neo Sensitabs, Rosco, Taastrup, Denmark) according to the National Committee for Clinical Laboratory Standards (CLSI) guidelines; for ciprofloxacin and AZI, minimal inhibitory concentration (MIC-values) were determined with the E-test macromethod (bioMérieux). Interpretation of results was performed according to the most recent CLSI guideline^50^. Isolates with intermediate susceptibility were considered resistant. MDR was defined as co-resistance to all three first-line antibiotics ampicillin, chloramphenicol and trimethoprim-sulfamethoxazole^15^. ESBL production was assessed by double disk method according to CLSI guidelines^50^. For ciprofloxacin, the susceptibility breakpoint of ≤0.064 mg L^−1^ was used; the term decreased ciprofloxacin susceptibility was used to indicate MIC values >0.064 mg L^−1^ and <1 mg L^−1^, and ciprofloxacin resistance was reserved for MIC values ≥1 mg L^−1^^50^. For AZI, isolates were considered resistant at MIC values ≥16 mg L^−1^^50^. Ethical approval for the Microbiological Surveillance was granted by the Institutional Review Board at the ITM in Antwerp, by the Ethics Committees of the Antwerp University (Belgium) and the School of Public Health (Kinshasa, DRC).

### Illumina and Pacbio whole-genome sequencing

DNA from all 81 strains was purified using the Gentra PureGene Yeast/Bact Kit (Qiagen, Hilden, Germany), following the manufacturer’s guidelines and DNA was sequenced on an Illumina HiSeq platform (Illumina, San Diego, USA). Illumina adapter content was removed from the reads using Trimmomatic v.0.33^51^. Public available Illumina sequencing data from 153*S*. Typhimurium strains originating from DRC, Mali, Nigeria, Uganda, Kenya, Malawi and Mozambique and a selection of 42 non-African genomes were included in the further genomics analysis^9–12,26^. This is a convenience sample including all publicly available African iNTS genomes. Strains are summarized in Supplementary Data 2.

A high-quality reference genome sequence of sublineage II.1 isolate 10433_3 was constructed using the PacBio RS II platform (PacBio, Menlo Park, CA, USA). The PacBio Template Prep Kit (PacBio, Menlo Park, CA, USA) and BluePippin™ Size Selection System protocol were used to prepare size-selected libraries (20 kb) from 5 μg of sheared and concentrated DNA. Sequencing was performed using the magnetic bead collection protocol, a 20,000 bp insert size, stage start, and 180-min movies. Sequence reads were assembled using HGAP v3 28 of the SMRT analysis software v2.3.0 (Pacbio, Menlo Park, CA, USA). The fold coverage to target when picking the minimum fragment length for assembly was set to 30 and the approximate genome size was set to 3 Mbp. The assembly was circularized using Circlator v1.1.3^52^. Finally, the circularized assembly was polished using the PacBio RS_Resequencing protocol and Quiver v1 of the SMRT analysis software v2.3.0^53^. Automated annotation was performed using PROKKA v1.11^54^ and genus specific databases from RefSeq^55^. The final assembled bacterial chromosome consisted of 4,877,289 bp, and two plasmid sequences of 274,695 bp and 94,649 bp.

### Read mapping, variant detection, and phylogenetic analysis

Illumina HiSeq reads were mapped to the *S*. Typhimurium reference genomes of ST313 lineage II (D23580, FN424405.1^56^) and ST313 sublineage II.1 (10433_3, ERZ1030005, this study) using SMALT v0.7.4 to produce a BAM file.

SMALT was used to index the reference using a kmer size of 20 and a step size of 13 and the reads were aligned using default parameters but with the maximum insert size set as 3 times the mean fragment size of the sequencing library. PCR duplicate reads were identified using Picard v1.92 (Broad Institute, Cambridge, MA, USA) and flagged as duplicates in the BAM file.

Variation detection was done using samtools mpileup v0.1.19 with parameters -d 1000 -DSugBf and bcftools v0.1.19^57^ to produce a BCF file of all variant sites, using the option to call genotypes at variant sites. The bcftools variant quality score was set to be greater than 50 (quality < 50), mapping quality was set to be greater than 30 (map_quality < 30), the allele frequency was required to be either 0 for bases called the same as the reference, or 1 for bases called as a SNP (af1 < 0.95), the majority base call was set to be present in minimal 75% of the reads mapping at the base (ratio < 0.75), the minimum mapping depth was four reads (depth < 4), at least two of these four had to map to each strand (depth_strand < 2), strand_bias was set to be less than 0.001, map_bias less than 0.001 and tail_bias less than 0.001. Otherwise, the base was called as uncertain and removed.

A pseudo-genome was constructed by substituting the base calls in the BCF file in the reference genome, uncertain sites were substituted with Ns. Insertions with respect to the reference genome were ignored, deletions were filled with N’s in the pseudo-genome.

Recombinant regions in the chromosome such as prophage regions and the *fljB* ORF in the chromosome were removed from the alignment and checked using Gubbins v1.4.10^58^. SNP sites were extracted from the alignment using snp-sites^59^ and used to construct a maximum likelihood phylogeny. RAxML v8.2.8^60^ with substitution model GTRCAT. Support for nodes on the trees was assessed using 1000 bootstrap replicates. A comprehensive tree, with reads mapped to ST313 lineage II (D23580, FN424405.1^56^) was rooted on *S*. Paratyphi A270 (ERR326600). Based on this tree, lineage II and sublineage II.1 isolates were identified and a high-resolution lineage II tree was constructed based on mapping to ST313 sublineage II.1 (10433_3, this study) and rooted to *S*. Typhimurium ST313 strain DT2. Trees were visualized using Figtree v1.4.2 and iTOL^61^. The comprehensive phylogenetic tree with spatiotemporal metadata, based on mapping against D23580 and rooted on *S*. Paratyphi A270 is made publicly available on MicroReact^62^ (https://microreact.org/project/xS5Xw6b3A).

### Bayesian phylogenetic analysis

We used BEAST2 v2.4.8^63^ to date evolutionary events, determine the substitution rate and produce a time-tree of African *S*. Typhimurium with tip-dates defined as the year of isolation (Supplementary Data 2). BEAUti xml’s were manually modified to specify the number of invariant sites in the genome. We employed a general time reversible (GTR) substitution model with gamma distributed rate heterogeneity. In addition, an uncorrelated log-normal relaxed molecular clock^64^ was used to model the variation of evolutionary (substitution) rates across branches. The extended Bayesian skyline plot (EBSP) demographic method^65^ was selected as this model does not depend on a prespecified parametric model of demographic history and the method has been proven to indicate the most appropriate demographic model for any given dataset. Earlier BEAST analyses on a related dataset identified the molecular clock model and tree prior used here to have the highest support by using Bayes factor (ratio of the marginal likelihoods of two competing models) based model selection^9^.

All parameters were estimated jointly in a BEAST2 analysis using 10 independent chains of 500 million MCMC generations, with samples taken every 50,000 generations. Log files were inspected in Tracer v1.6 for convergence, proper mixing, and sufficient sampling, assessed by whether the chain length produced an effective sample size (ESS) for all parameters larger than 300. A 30% burn-in was removed from each run, and LogCombiner v2.5.0 was used to combine log and tree files of the independent BEAST2 runs. Parameter medians and 95% highest posterior density (HPD) intervals were finally estimated from 70,000 sampled MCMC generations. The entire analysis was replicated on five random subsets of 100 taxa of the dataset to test if the results were affected by sampling bias.

The entire analysis was run while sampling only from the prior, to ensure that prior parameters were not causing over-constraining of the calculations. The resulting parameter distributions were compared in Tracer. The posterior sample of the time-trees were summarized in TreeAnnotator v2.5.2 to produce a maximum clade credibility tree with the posterior estimates of node heights visualized on it.

### Genomic rearrangements and SNP analysis

Large genomic rearrangements were identified by analysis of the aligned reference genome sequences using the Artemis Comparison Tool (ACT).

A VCF file containing all SNP sites was extracted using snp-sites^59^ from all strains included in this study. Conserved SNPs between lineage II and lineage II.1 isolates were extracted using custom calculations in R v.3.3.3, thereby comparing SNPs with the D23580 reference sequence and annotation (FN424405.1). SNPs that were conserved in 51 of the 53 sublineage II.1 strains, and not present in lineage II strains were subjected to a functional analysis. These SNPs were functionally studied by comparison with the D23580 annotation^56^. Ortholog loci in *S*. Typhimurium LT2 (NC_003197.2) were identified using BLASTN 2.6.0+.

For SNPs present in coding regions, the effect of the SNP on the respective amino acid sequence was assessed. For non-synonymous SNPs, the PAM1 value and delta bitscore value was included. The higher the PAM1 value, the more frequent specific amino acid substitutions are observed. Delta bitscores were calculated by subtracting the bitscore for a given HMM domain in lineage II from the bitscore of the orthologous domain lineage II.1 strains^66^.

To calculate the invasiveness index per strain, sequence reads of each sample were mapped against the *S*. Typhimurium SL1344 reference genome (FQ312003.1) using BWA mem v0.7.12^67^ to produce a BAM file thereby using default parameters but maximum insert size changed to 3 times the mean fragment size of the sequencing library. We used Picard (http://broadinstitute.github.io/picard) to identify optical duplicates generated during library preparation. SNPs were called using samtools v1.2 mpileup^68^, variants with coverage < 10 or quality < 30 were filtered and excluded. SNP calls were used to produce variant coding sequences. Protein sequences were then screened using phmmer to identify the closest homologs to the 196 predictive genes used by the invasiveness index model. These genes were then scored against profile hidden Markov models (HMMs) for these protein families from the eggNOG database to test for uncharacteristic patterns of sequence variation. Bitscores produced in the comparison of each protein sequence to its respective protein family HMM were then used as input to the model. The groups were compared using a Mann Whitney *U-*test.

### Sequence typing, resistance, and plasmid analysis

Resistance genes were determined from the raw Illumina sequencing data using ariba v 2.11.1^69^ with CARD database version 1.1.8^70^. SNPs explaining a DCS phenotype was checked individually for strains showing DCS. The *gyrA*, *gyrB*, *parC*, *parE*, *acrB* sequences of reference sequence from *S*. Typhimurium LT2 (NC_003197.2) were compared to assemblies of the DCS strains using BLASTN 2.6.0+. The presence of plasmid replicons was determined from raw Illumina reads using SRST2^71^ with the complementary P18 plasmid replicon database.

The PacBio reference sequence of plasmid pSTm-ST313-II.1 (*S*. Typhimurium DRC, this study) was searched for similar nucleotide sequences publicly available using NCBI Blast with the Nucleotide collection (nt), update 2017/07/13. Pairwise comparisons were done using BRIG^72^. pSTm-ST313-II.1 was compared with plasmids R478 (*Serratia marcescens* USA, BX664015), pKST313 (*S*. Typhimurium, LN794248) and pSTm-A54650 (*S*. Typhimurium, LK056646). A de novo assembly was performed based on Illumina sequences of isolate 5390_4 (This study)^73^ and the assembly of the plasmid sequence was used for pairwise comparative analysis. Hereto, the contigs of the assembly were reordered using Mauve version 2015_02_25^74^ and the 12 contigs homologous to pSTm-ST313-II.1 were retained for further analysis.

### Flagella expression

Presence of *fljB* (FljB) and *fliC* (FliC) were confirmed at the DNA and protein level for a *S*. Typhimurium reference lineage II (isolate 9412_3) and sublineage II.1 (10433_3) strain. DNA was extracted from both strains using Gentra Puregene Yeast/Bact. Kit (Qiagen, USA) and PCR-amplified using *fljB* (fljB-F and fljB-R) and *fliC* (fliC-F and fliC-R) specific primers (Supplementary Table 5).

For protein samples, overnight cultures were centrifuged at 1500 g and pellets were suspended in 300 µL phosphate-buffered saline (PBS, pH 7.4). The suspension was homogenized using FastPrep-24 (MP Biomedicals, Santa Ana, California, USA) and centrifuged at 15,000 rpm. 10 µL of the supernatant was diluted 1:1 with sample buffer (Laemmle, Sigma-Aldrich, St. Louis, Missouri, US) before loading on SDS-page gel (12%). After running (45 min 100 V) the gel was coloured with Coomassie blue.

### Biofilm assays

Strains were grown in 10 ml low salt LB Broth overnight, diluted 1:100 in PBS and 5 µL was spotted onto rdar phenotype plates (1.5% Agar, 1% Tryptone, 0.5% Yeast Extract, 20 µg mL^−1^ Coomassie Brilliant Blue, 40 µg mL^−1^ Congo Red in water)^75^. The plates were incubated at 27 °C without inversion for 84 h, phenotype analysed and photographed. Colonies were quantitatively analysed using the IRIS software^76^.

### Biolog assays

Four *S*. Typhimurium ST313 lineage II (12299_3, 9266_3, 9412_3 and D23580) and four *S*. Typhimurium sublineage ST313 sublineage II.1 (10393_3, 10433_3,12306_3 and 8866_3) isolates were analysed on the OmniLog phenotype MicroArray (PM) platform, in three independent biological replicates each. Overnight cultures cells were transferred with a sterile swab to 15 mL IF-0 (12.5 mL IF-0 with 2.5 mL H_2_O) and stirred to obtain a uniform suspension. Turbidity of the suspension was adjusted to 42% transmittance T by using the Biolog Turbidimeter. Plates PM 1 and PM 2 were inoculated with 100 µL per well of the cell suspension mix diluted 1:5 in IF-0 containing 1.2% dye mix A. All plates were covered by anaerobic sealing foil (Roche) and incubated in the Omnilog phenotype MicroArray system for 48 h at 37 °C. Statistical calculations and visualization were done in R (R Core Team) by using the opm package^77^. The curve parameters were estimated using spline fitting. The mean of the area under the curve (AUC) was compared between sublineage II.1 versus lineage II strains using a linear model based on a Tukey-type contrast using the opm_mcp function from the multcomp package^78^.

### Mutant construction

Mutant strains *S*. Typhimurium D23580 Δ*asmA*, *S*. Typhimurium D23580 Δ*yhjJ*, *S*. Typhimurium D23580 Δ*yhjK* and *S*. Typhimurium D23580 Δ*wxzC* were constructed. Hereto, the Kanamycin resistance gene (*kan*) was PCR-amplified from pKD4 using Q5 HotStart DNA Polymerase (New England Biolabs, Massachusetts, U.S.A.) and gene specific oligos (Integrated DNA Technologies, Illinois, USA) for *asmA* (asmA-F and asmA-R), *wzxC* (wzxC-F and wzxC-R), *yhjJ* (yhjJ-F and yhjJ-R) and *yhjK* (yhjK-F and yhjK-R), listed in Supplementary Table 5. DNA was prepared by gel extraction (Qiagen, Hilden, Germany) and ethanol precipitation.

The pSIM18 vector carrying the lambda RED recombinase system^79^ was electroporated into *S*. Typhimurium D23580 using 10.5 ng DNA at a setting of 2.5 kV, 200 ohms and 25 µF (Bio-Rad MicroPulser, California, USA). 750 µL of SOC outgrowth medium (Thermo Fisher Scientific, Massachusetts, USA) was added and cells grown at 30 °C for 90 min, 100 μL aliquots were plated out onto plates containing Hygromycin and colonies recovered next day. The subsequent *S*. Typhimurium D23580::pSIM18 was used to create the mutant strains.

*S*. Typhimurium D23580::pSIM18 was grown overnight at 30 °C and then diluted 1 in 100 the next day in low salt LB plus 100 µg mL^−1^ Hygromycin B (Thermo Fisher, Massachusetts, U.S.A.) and incubated for 3 h, shaking at 30 °C to an optical density (OD) of 0.4. The culture was heat-shocked at 42 °C for 15 min, cooled on ice for 10 min and then was spun for 10 min at 2500×*g* and washed twice in ice cold 10% glycerol. The cell pellet was finally resuspended in 140 µL of 10% glycerol and 60 µL aliquots were electroporated with 1 µg of DNA (Table 1) in a precooled 2 mm electroporation cuvette. Cells were then incubated in 500 µL of pre-warmed SOC outgrowth medium (Thermo Fisher Scientific, Massachusetts, USA) at 37 °C for 2 h. 3 × 100 µL aliquots were plated onto 50 µg mL^−1^ Kanamycin (Thermo Fisher, Massachusetts, USA) LB plates and grown overnight at 37 °C. The remaining volume was left at room temperature overnight and plated on 50 µg mL^−1^ Kanamycin and left to grow at 37 °C. The knock-out mutation was confirmed using Illumina sequencing.

### THP-1 macrophage invasion and replication assay

THP-1 monocytes (European Collection of Authenticated Cell Cultures (ECACC), cat no. 88081201) were plated at 1 × 10^5^ cells per well in RPMI (Gibco) with 2 mM L-glutamine (Sigma) and 10% foetal bovine serum (Sigma) with PMA (Phorbol 12-myristate 13-acetate, (Sigma) to differentiate to macrophages. After 72 h, media was changed to the supplemented RPMI (as defined above) for 24 h before infection. Bacterial isolates SL1344, D23580, 2101, 9266_3, 12299_3, 6948_3, 9412_3, 10055_3, 8866_3, 12155_3, 10393_3, and 10433_3 were inoculated in 10 mL low-salt lysogeny broth (LB) from frozen stocks and incubated at 37 °C, shaking for ~17.5 h prior to use. For infection, bacteria were diluted in PBS to an OD of 1 before adding to RPMI. 500 µL of bacteria in media were added to cells for a multiplicity of infection of 20:1 and incubated at 37 °C for 30 min. At 30 min, media containing bacteria was removed and replaced with media containing 0.1 mg mL^−1^ gentamicin. For the 1 h invasion timepoint, at 1 h post-infection, cells were washed 1x in PBS then lysed in 1% Triton, and serial dilutions at 10^−1^, 10^−2^, 10^−3^ prepared in PBS. 50 µL of each dilution was spotted on LB agar plates in 5 technical replicates of 10 µL spots and incubated at 37 °C. For the 6 h intracellular replication timepoint, gentamicin-containing media was replaced by the supplemented RPMI at 1 h post-infection and incubated at 37 °C for another 5 h. At 6 h post-infection, cells were washed 1× in PBS and lysed with 1% Triton, as for the cells at the 1 h timepoint. Agar plates were incubated until colony forming units were visible for counting to calculate viable bacteria per mL. Experiments were done in three biological replicates, mean and standard error of the mean was calculated for all strains.

### Mouse infection

*Salmonella* strains were grown static overnight in LB at 37 °C. Inoculations were prepared by diluting overnight culture in PBS. Inoculums were plated post infection on LB agar plates for confirmation. Groups of C57/Bl6 mice were pre-treated with 50 mg of Streptomycin by oral gavage and infected intravenously with 2 × 10^2^ colony forming units (CFU) or orally with 2 × 10^6^ CFU with each *Salmonella* strain. At day 4 post infection mice were culled and organs removed. Homogenised organs were serially diluted in PBS and 20 µL of each dilution were spotted into LB agar plates. CFUs were counted next day to calculate number of CFUs per organ. All experiments on animals were performed under a UK animal license that has been through local ethical review before being approved by Sanger—Animal Welfare and Ethical Review Body (AWERB) and performed according to the regulations of the UK Home Office Scientific Procedures Act (1986).

### Material availability

Requests for obtaining biological material (mutant strains) should be addressed to the corresponding author. Exchange of clinical isolates should always be in agreement with the ITM (Antwerp, Belgium) and INRB (Kinshasa, DRC), co-owning the bacterial isolates.

### Reporting summary

Further information on research design is available in the Nature Research Reporting Summary linked to this article.

## Supplementary information

Supplementary Information

Reporting Summary

Description of Additional Supplementary Files

Supplementary Data 1

Supplementary Data 2

Supplementary Data 3

Source Data

## Data Availability

Sequence data that support the findings of this study have been deposited in SRA with accession codes as listed in Supplementary Data 1. The annotated Pacbio’d reference genome of *Salmonella* Typhimurium strain 10433_3 is uploaded to GenBank with accession ID ERS1310131. The generated phylogenetic tree (as presented in Fig. 2a) is publicly accessible in MicroReact: https://microreact.org/project/ZfwCFTj2-/d9f0ce6f. The source data underlying Fig. 6a, b and Supplementary Figs 6, 9–11 are provided as Source Data File. All other datasets generated during and/or analysed the current study are available from the corresponding author on reasonable request.
