# Supplementary material for: An African Salmonella Typhimurium ST313 sublineage with extensive drug-resistance and signatures of host adaptation
Source: Nat Commun. 2019 Sep 19;10:4280. doi: 10.1038/s41467-019-11844-z (PMC6753159; doi:10.1038/s41467-019-11844-z)
Supplement: Supplementary file 1 — Supplementary Information [file 41467_2019_11844_MOESM1_ESM.pdf]

Supplementary information

1. Supplementary Note 1: Sample selection for this study

This study describes a novel sublineage of extensive drug-resistant (XDR) *Salmonella enterica* serovar Typhimurium (*S.* Typhimurium) in the Democratic Republic of the Congo (DRC), of which the isolates were identified during an ongoing bloodstream surveillance program in DRC [1, 2, 3]. Samples originating from gastroenteritis are not included in the surveillance program.

Index isolates: All available azithromycin resistant isolates obtained during the period 2008-2016 were included in the analysis (n = 54). All but four index isolates were recovered from HGR Kisantu in DRC. Thirty-six of these isolates are part of the surveillance analysis described in Lunguya et al. (2013) [3] and Kalonji et al. (2015) [1]. A breakdown of these isolates is given in Table 1. Of the 54 index isolates, 51 show XDR.

Table 1: Breakdown of index isolates included in this study

Year	Kinshasa	Bas-Congo	Orientale	Grand Total
2008	1			**1**
2013	1	3		**4**
2014		31	1	**32**
2015		11		**11**
2016		5	1	**6**
**Grand Total**	**2**	**50**	**2**	**54**

Control isolates: A total of 27 *S.* Typhimurium were selected as controls– 22 of them were MDR, as nearly 90 % of the *S.* Typhimurium isolates of the surveillance were MDR [1, 3]. These control isolates were selected to provide local context to our genomics analysis and to cover the spatiotemporal variation in the surveillance isolates. They were isolated between 2007 and 2016. Seventeen of them have been described as part of previous studies [1, 3]. Of note, indications for sampling were consistent over place and time, as was apparent from the proportion of pathogens isolated, but surveillance sites were not consistently active because of stock ruptures, staff movements, funding and insecurity. A breakdown of these isolates is given in Table 2, displaying the distribution: the numbers selected as controls and the total numbers of non-duplicate *S.* Typhimurium isolates recovered are given.
This control group was a representative sample of the DRC surveillance.

Table 2: Breakdown of control isolates included in this study

Year	Kinshasa		Bas Congo		Equateur		Orientale		Grand Total	
	C	T	C	T	C	T	C	T	C	T
2007	**1**	14	**1**	0		0		0	**2**	14
2008	**2**	24	**1**	26		14		3	**3**	67
2009		4	**2**	36		5		7	**2**	52
2010		4	**1**	31		4		4	**1**	43

2011		4	**2**	26	**1**	38		5	**3**	73
2012		4	**2**	13		26		13	**2**	56
2013		4	**2**	44		2		14	**2**	64
2014		2	**6**	145		13		28	**6**	188
2015		0	**3**	160		3		4	**3**	167
2016		0	**2**	239		2	**1**	9	**3**	250
**Grand Total**	**3**	60	**22**	720	**1**	107	**1**	87	**27**	974

The number of control (C) isolates are given in bold per province in the Democratic Republic of the Congo and per year. The total (T) number of isolates originating from the surveillance in the respective provinces and years are indicated.

Context isolates: For an optimal genetic context we used sequencing data that was available from existing databases: 153 African [4, 5, 6, 7, 8] and 42 non-African [8] *S.* Typhimurium whole genome sequences to the dataset. The complete list of 276 analysed *S.* Typhimurium genomes with the year, place and source of isolation is presented in Supplementary Table 2. This context dataset includes non-ST313 isolates and isolates associated with gastroenteritis.
This context group was a convenience sample

Supplementary Figures

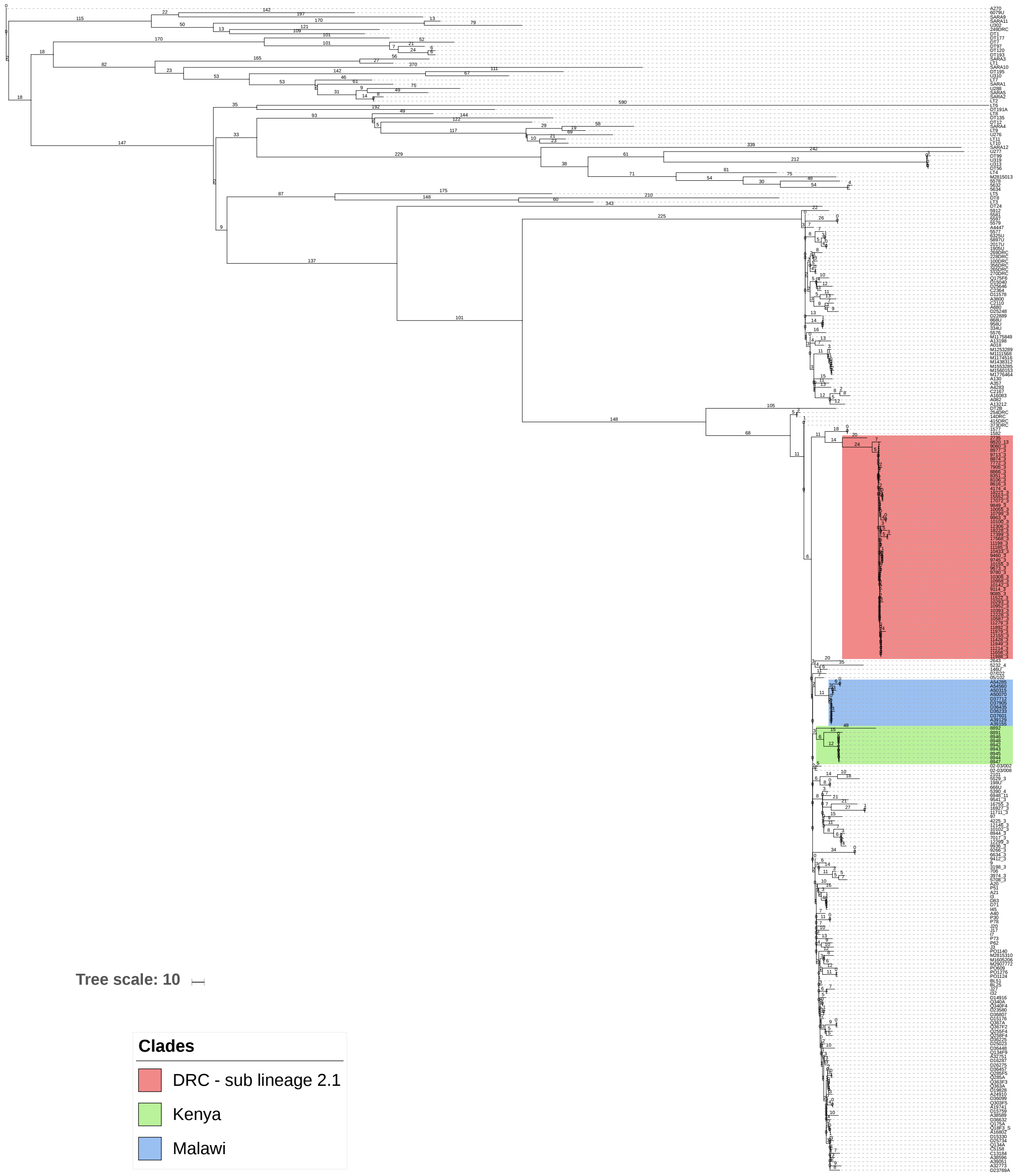

Supplementary Figure 1: Phylogenetic tree with annotated SNP distances

Maximum likelihood phylogenetic tree with African and non-African *Salmonella* Typhimurium strains, based on mapping to *Salmonella* Typhimurium ST313 lineage II reference strain D23580 [9]. Numbers of conserved SNPs are annotated per branch. Sublineage II.1 from the Democratic Republic of the Congo (this study), and clonal outbreaks from Kenya and Malawi are annotated in colour [4, 10].

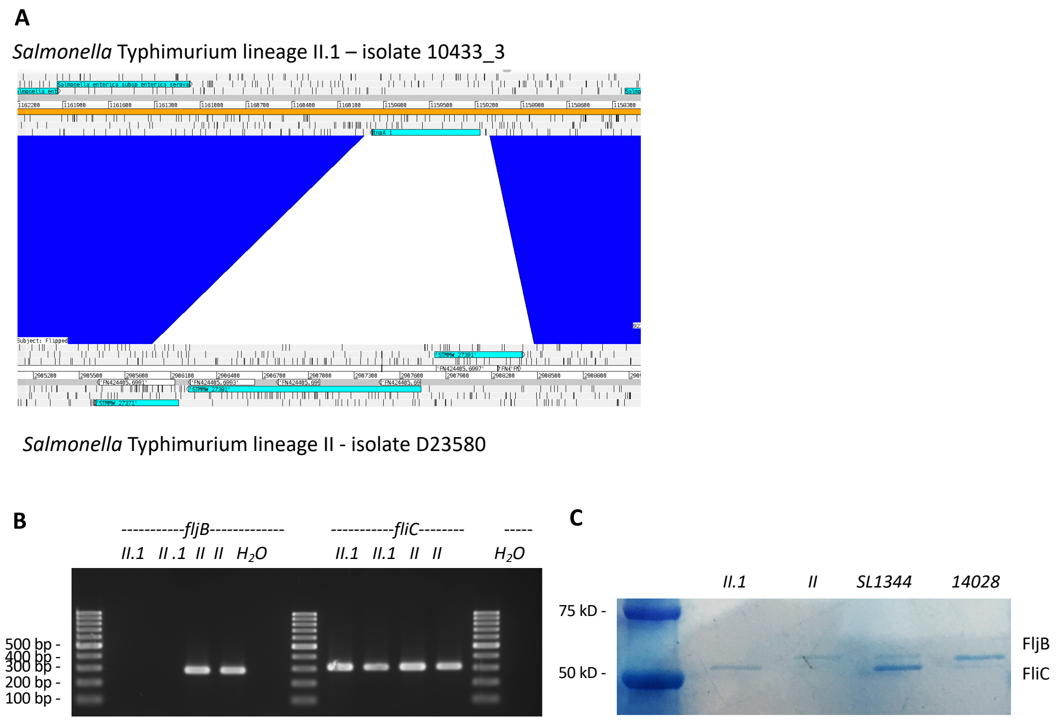

Supplementary Figure 2: Loss of *fljB* gene and FljB protein in sublineage II.1

A. Genomic comparison of reference genomes from *Salmonella* Typhimurium sublineage II.1 isolate 10433_3 (this study) and *Salmonella* Typhimurium lineage II isolate D23580 (FN424405) using ACT software. A region of 2505 bp including STMW_27391 (DNA invertase *hin*), STMW_27381 (flagellin gene *fljB*) and part of STMW_27371 (repressor of phase 1 flagellin *fljA*) was replaced by a region of 820 bp including the *tnpA_1* gene. **B.** Presence of *fljB* and *fliC* genes after PCR-amplification with gene specific primers in lineage II.1 (10433_3) and lineage II (9412_3) isolates. **C.** Western blot of purified flagellae in lineage II.1 (10433_3), lineage II (9412_3) isolates, and *Salmonella* Typhimurium SL1344 and 14028 isolates.

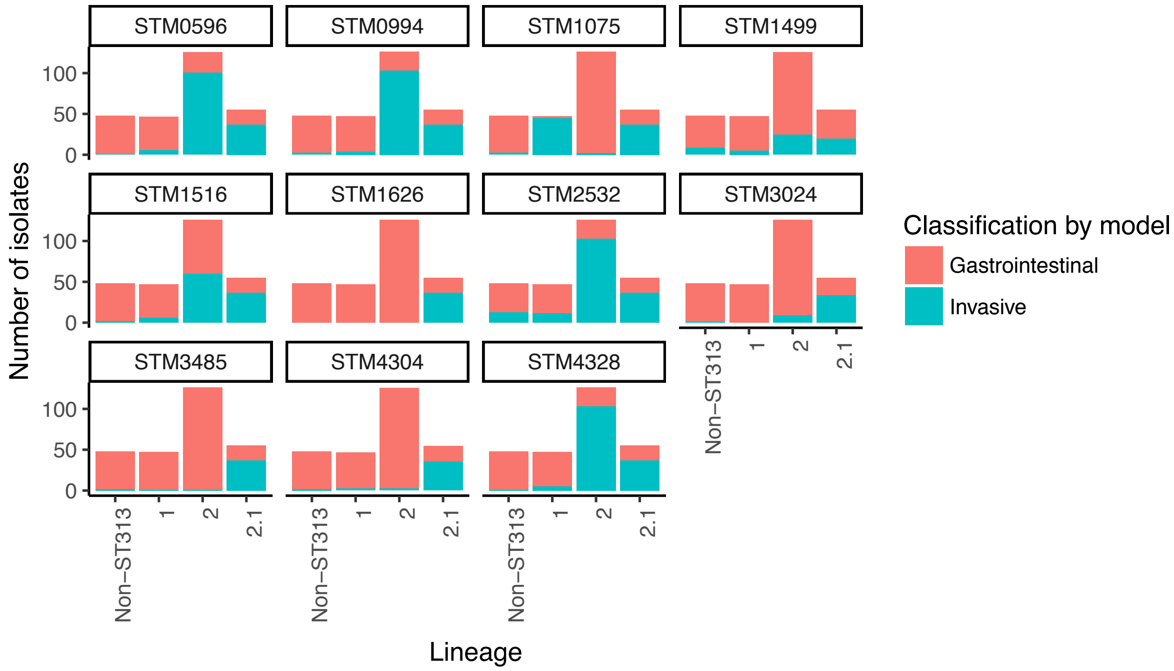

Supplementary Figure 3: Counts of isolates from each lineage carrying gene copies more characteristic of an invasive (green) or gastrointestinal (red) lineage.

Of the 196 genes used in the model to calculate the invasiveness index, genes contributing to an increased invasiveness index of sublineage II.1 are STM0596, STM0994, STM1075, STM1499, STM1516, STM1626, STM2532, STM3024, STM3485, STM4304, and STM4328. These genes show an increase in mutations in sublineage II.1 that caused the gene variants to be classified as more characteristic of an invasive strain, likely due to the accumulation of deleterious mutations.

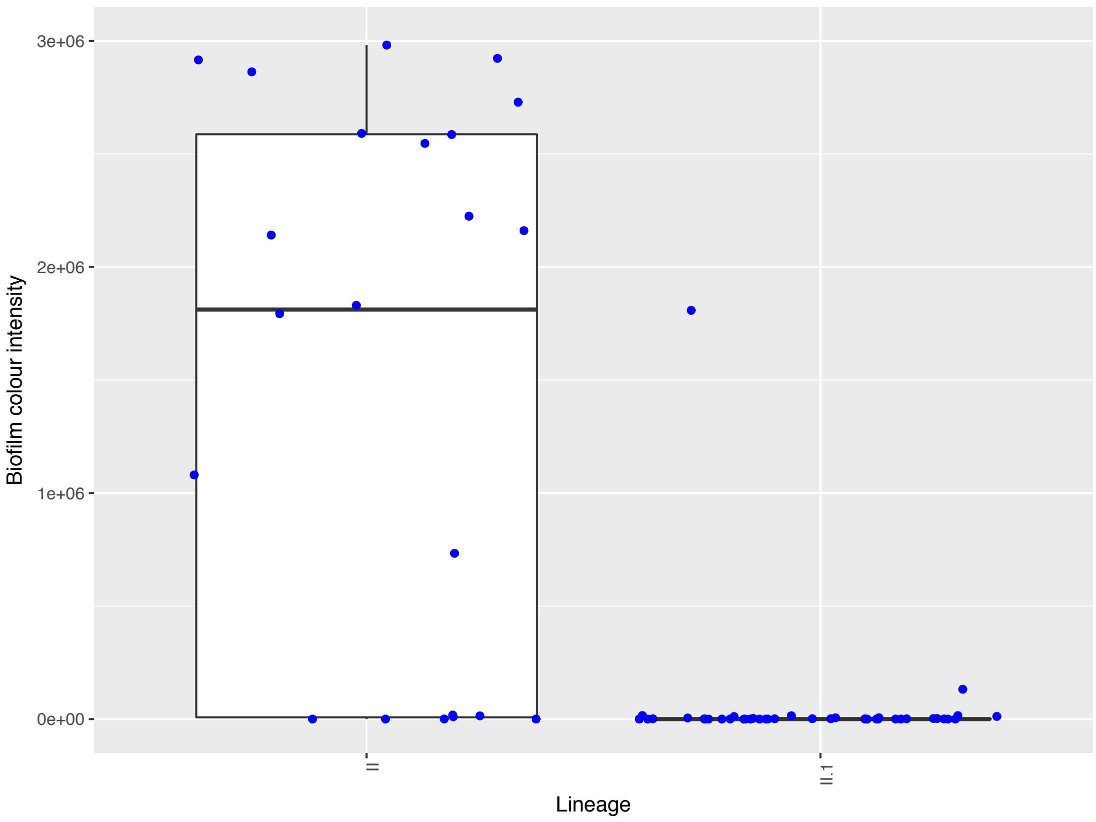

Supplementary Figure 4: Biofilm formation of *Salmonella* Typhimurium ST313 isolates

Colour intensities of red, dry and rough (rdar) morphotype [11] of the 81 *Salmonella* Typhimurium ST313 isolates from the Democratic Republic of the Congo included in this study. All isolates are grown as rdar morphotypes and analysed using IRIS software [12]. The values for the biofilm colour intensity as calculated with IRIS, is plotted per isolate. All sublineage II.1 show a low colour intensity, except isolate 2735. Lineage II isolates show a higher mean and higher variation in the colour intensity. Boxplot centre lines represent median values, box limits present upper and lower quartiles; whiskers the 1.5 interquartile range and blue points the individual observations. Source data are provided as a Source Data file.

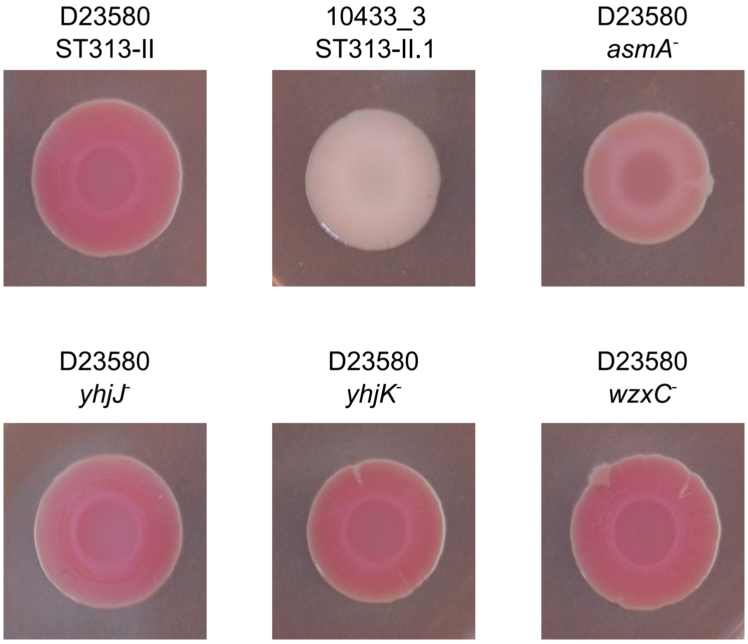

Supplementary Figure 5: Biofilm formation of mutant strains

Red, dry and rough (RDAR) morphotype [11] of *Salmonella* Typhimurium ST313 lineage II strain D23580 [9], *Salmonella* Typhimurium ST313 lineage II.1 strain 10433_3 (this study) and *Salmonella* Typhimurium ST313 lineage II D23580 *asmA, yhjJ, yhjK* and *wzxC* knock-out strains (this study). Knock-outs in *yhjJ, yhjK* and *wzxC* did not show a different phenotype compared to mother strain D23580. A knock-out in *asmA* showed a reduced colour intensity intermediate to lineage II mother strain D23580 and sublineage II.1 strain 10433_3.

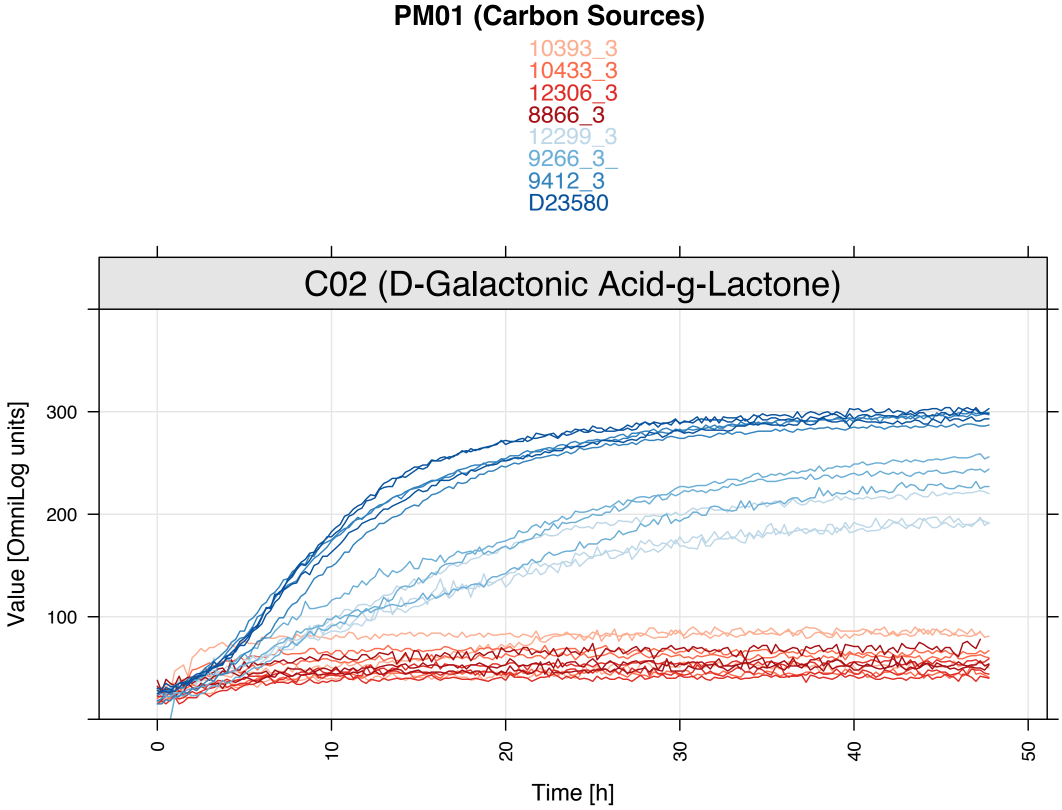

Supplementary Figure 6: Growth on D-Galactonic Acid-g-Lactone

Respiration of *Salmonella* Typhimurium ST313 lineage II (D23580, 9412_3, 9266_3 and 12299_3) and four *Salmonella* Typhimurium sublineage ST313 sublineage II.1 (10393_3, 10433_3, 12306_3 and 8866_3) isolates using the OmniLog phenotype MicroArray (PM) platform on D-Galactonic Acid-g-Lactone, in three biological replicates each.

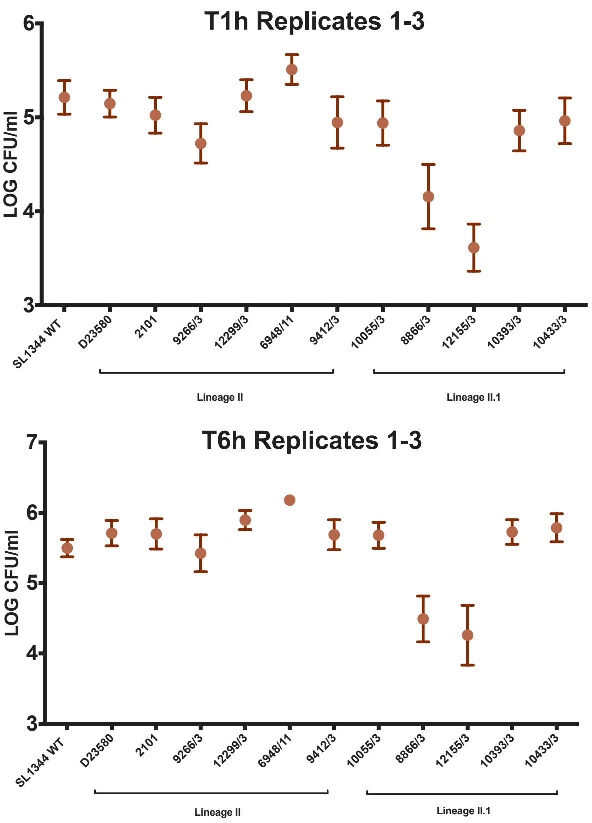

Supplementary Figure 7: THP-1 Macrophage infection

THP-1 monocytes were differentiated to macrophages and subjected to infection of single *Salmonella* Typhimurium strains. After incubation of 30 mins, cells were treated with gentamicin. Intracellular bacterial counts were determined at 1 h and 6 h post-infection. Experiments were done in three biological replicates. The mean and standard error of the mean of three biological replicates are plotted. Source data are provided as a Source Data file.

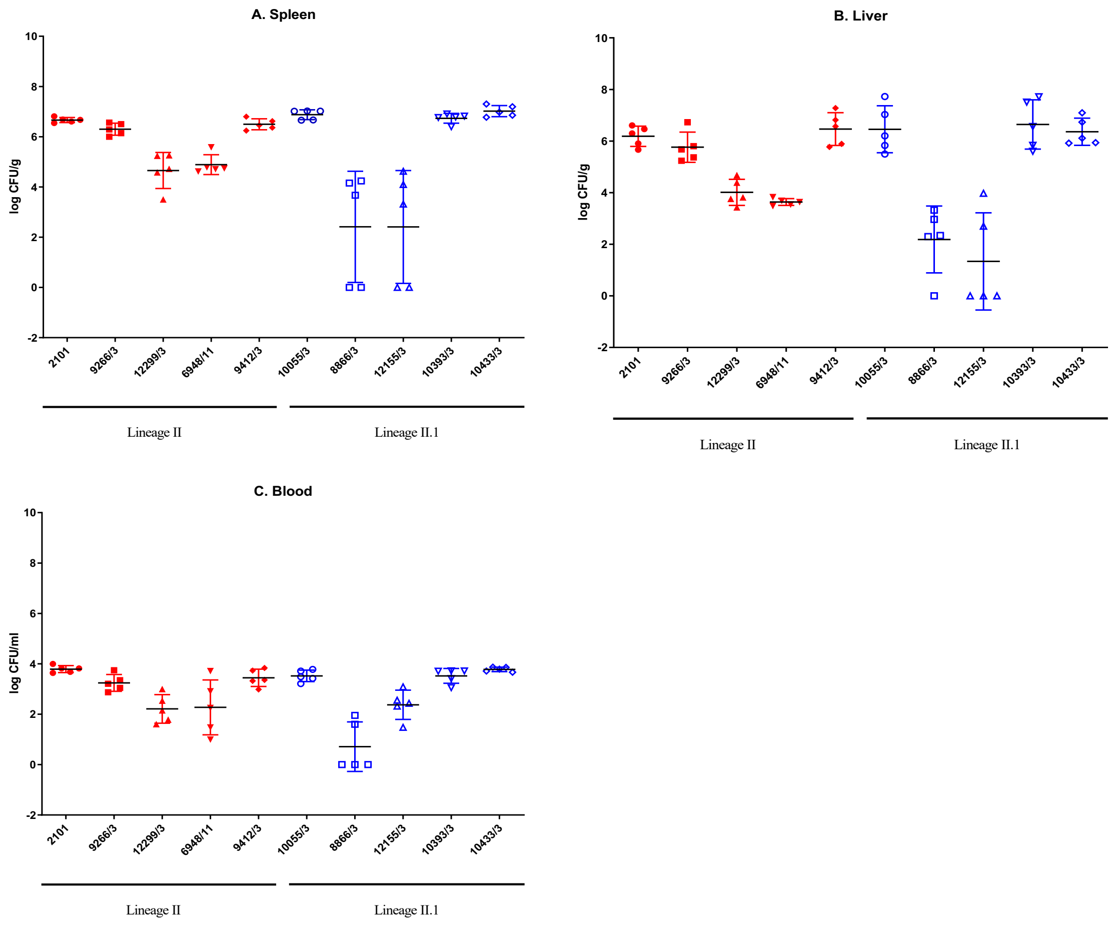

Supplementary Figure 8: Intravenous mice infections

Mice (n=5 per group) were infected intravenously with 2 x 10^2 cfu for each individual strains of *Salmonella* Typhimurium from both linage II and linage II.1. The graphs show bacterial counts per gram of tissue from the spleen (A) and liver (B) and per ml of blood (C) at day 4 post infection. The mean and standard deviation of the mean of 5 infections are plotted. Source data are provided as a Source Data file.

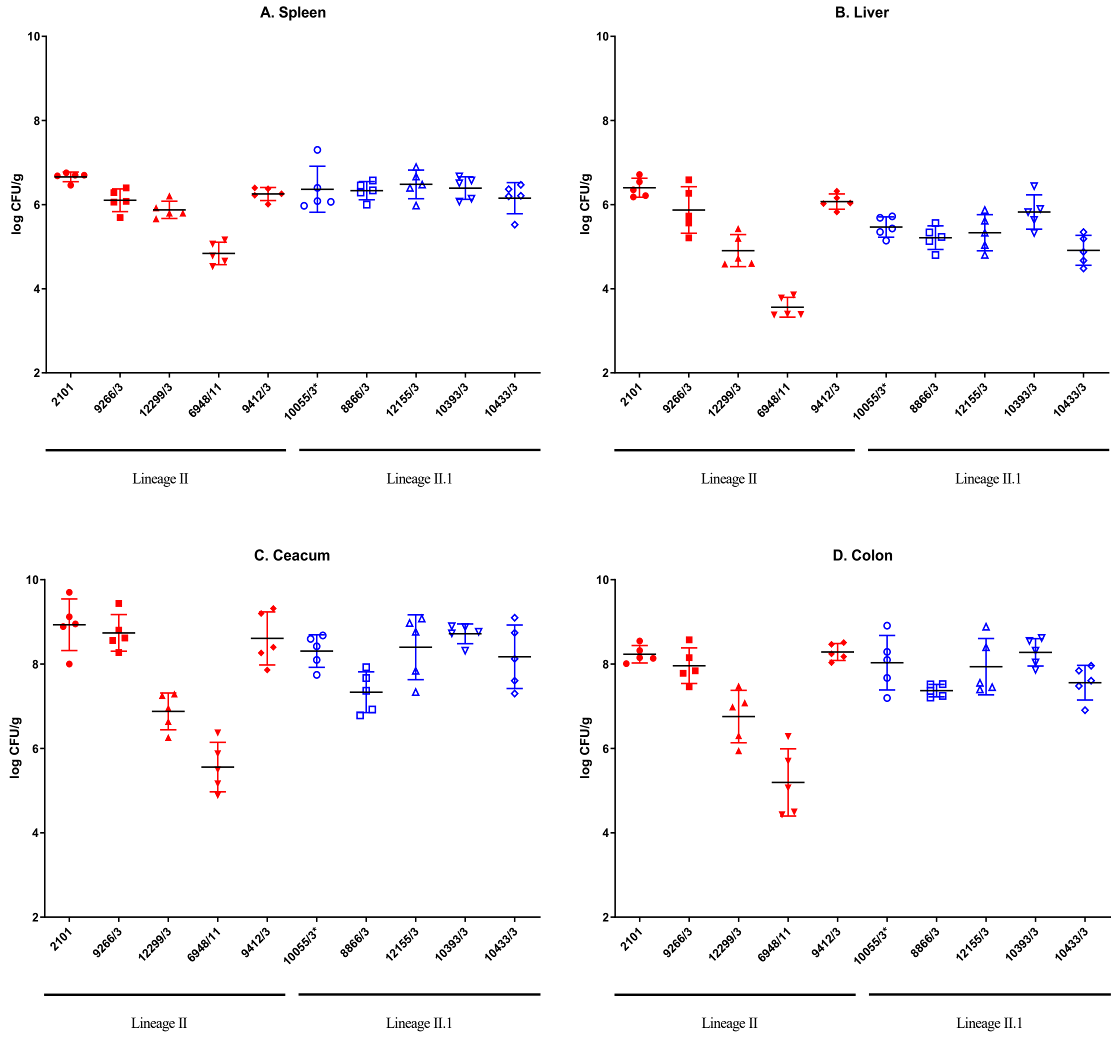

Supplementary Figure 9: Oral mice infections

Mice (n=5 per group) were infected by oral gavage with $2x10^6$ for each individual strains of *Salmonella* Typhimurium from both linage II and linage II.1. The graphs show bacterial counts per gram of tissue from the spleen (A) and liver (B), caecum (C) and colon (D) at day 4 post infection. The mean and standard deviation of the mean of 5 infections are plotted. Source data are provided as a Source Data file.

2. Supplementary Tables

Supplementary Table 1: IncHI2 plasmids from *Salmonella* Typhimurium ST313

Name	Host	Origin	Year	Accession ID	Length	Reference
Complete plasmid sequences						
pSTm-ST313-II.1	*S.* Typhimurium	DRC, Kisantu	2014		274695 bp	This study
pKST313	*S.* Typhimurium	Kenya	2009-2012	LN794248	300375 bp	Kariuki 2015 [10]
pSTm-A54650	*S.* Typhimurium	Malawi	2009	LK056646	309406 bp	Feasey 2014 [4]
R478	*Serratia marcescens*	USA	1969	BX664015	274762 bp	Gilmour 2004 [13]
Plasmid assembly draft						
pSTm-5390_4	*S.* Typhimurium	DRC, Kisangani	2016		251955 bp	This study

Supplementary Table 2: Non-synonymous SNPs acquired in sublineage II.1 versus lineage II

SNP-POS	Strand	Gene	LT2 Locus	Length	Ratio	ST313-2 AA	ST313-2.1 AltAA	PAM1 score	Domain	DBS	II
275249	+	*chiA*	STM0233	1764	0.61	E	G	7			
517652	-	*ispA*	STM0423	900	0.30	M	I	12	polyprenyl_synt	1.9	
1024714	-	*yhhw*	STM0951	861	0.50	T	N	9			
1164658	+	*helD*	STM1075	2055	0.75	A	V	18			Yes
1543952	-	*rstB*	STM1471	1302	0.21	A	T	32			
1545332	-	*SBOV14671*	STM1472	1701	0.41	D	E	53	Amidohydro_3	-0.9	
1709552	-	*trg*	STM1626	1626	0.29	M	I	5	TarH	2.6	Yes
1751279	+	*NA*	STM1665	687	0.33	T	N	13	B3_4	0	
2130288	-	*cbiH*	STM2027	726	0.13	G	D	11	TP_methylase	2.8	
2146426	+	*pduH*	STM2044	351	0.28	G	C	1	Dehydratase_MU	4.8	
2207729	-	*wzxC*	STM2102	1479	0.92	G	S	21			
2229128	-	*asmA*	STM2120	1857	0.74	T	M	6	AsmA	3	
2547741	-	*mntH*	STM2408	1242	0.71	V	L	11	Nramp	-0.9	
2913876	+	*iroC*	STM2774	3654	0.91	G	C	1	ABC_tran	5.2	
3055657	-	*mexR*	STM2920	405	0.85	G	R	1			
3691796	+	*yhgH*	STM3510	684	0.04	L	P	3			
3767745	+	*tcp*	STM3577	1644	0.02	I	F	7	TarH	-0.4	
3815074	-	*yhjK*	STM3615	2007	0.76	P	L	2	EAL	2.6	
4573338	-	*dcuS*	STM4304	1632	0.54	V	I	57	PAS	-0.6	Yes

A summary is given of the non-synonymous SNPs present in at least 51 of the 53 sublineage II.1 isolates and not present in all lineage II strains from this study. SNPs were annotated based on the *Salmonella* Typhimurium D23580 annotation [9]. Ortholog loci in *Salmonella* Typhimurium LT2 were identified using BLASTN 2.6.0+. The full length (in bp) of the gene harbouring the SNP is given, and the ratio where the SNP is located in relation to the full length of the gene. The PAM1 value and delta bitscore (DBS) value is included. The higher the PAM1 value, the more frequent specific amino acid substitutions are observed. Delta bitscores were calculated by subtracting the bitscore for a given HMM domain from the bitscore of the orthologous domain in lineage II versus lineage II.1 isolates [14]. SNPs that were contributing in an increased invasiveness index (II) are indicated.

Supplementary Table 3: Synonymous SNPs acquired in sublineage II.1 versus lineage II

SNP-POS	Strand	Gene	LT2 Locus	Length	Ratio
1159181	-	*sulA*	STM1071	510	0.77
1326641	-	*envE*	STM1242	522	0.55
1455754	-	*lppB*	STM1376	303	0.39
1543953	-	*rstB*	STM1471	1302	0.21
2020792	-	*uvrC*	STM1946	1833	0.53
2332017	+	*setB*	STM2207	1182	0.54
3475715	-	*rbfA*	STM3285	402	0.25
3663385	-	*damX*	STM3485	1278	0.52
3811877	-	*yhjJ*	STM3613	1488	0.71
4371170	+	*btuB*	STM4130	1845	0.17
4576847	+	*dmsA*	STM4305	2430	0.93
4807734	+	*NA*	STM4528	288	0.38

A summary is given of the synonymous SNPs present in at least 51 of the 53 sublineage II.1 isolates and not present in all lineage II strains from this study. SNPs were annotated based on the *Salmonella* Typhimurium D23580 annotation [9]. Ortholog loci in *Salmonella* Typhimurium LT2 were identified using BLASTN 2.6.0+. The full length (in bp) of the gene harbouring the SNP is given, and the ratio where the SNP is located in relation to the full length of the gene.

Supplementary Table 4: SNPs in non coding regions acquired in sublineage II.1 versus lineage II

	Upstream			Downstream		
**SNP-POS**	**Strand**	**Gene**	**LT2 Locus**	**Strand**	**Gene**	**LT2 Locus**
442214	+	*NA*	STM0355	+	***nanT***	**STM0356**
858803	+	*ybgF*	STM0750	+	***nadA***	**STM0756**
1014500	**-**	***virK***	**STM0940**	+	***macA***	**STM0941**
1341768	**-**	***ynaI***	**STM1260**	+	***NA***	**STM1261**
1346289	**-**	***ariR***	**STM1267**	+	***aroQ***	**STM1269**
1509413	**-**	***gloA***	**STM1435**	-	*nemA*	STM1436
2871020	+	*prsE*	STM2692	+	***intA***	**STM2740**
3839017	**-**	***dppA***	**STM3630**	-	*ygfU*	STM3631
4298205	+	*ushB*	STM4064	+	***yicJ***	**STM4065**

A summary is given of the SNPs present in the intergenic regions of at least 51 of the 53 sublineage II.1 isolates and not present in all lineage II strains from this study. Neighbouring genes are indicated and annotated based on the *Salmonella* Typhimurium D23580 annotation [9]. Ortholog loci in *Salmonella* Typhimurium LT2 were identified using BLASTN 2.6.0+.

Supplementary Table 5 – Primers used in this study

Name	Nucleotide sequence
fljB-F	GCTCCTGTCGCTTCATCGTA
fljB-R	ACGGTACAGTAACCCTTGCG
fliC-F	AACAGATGCTGTGCCGGTAA
fliC-R	CTCGGCTACTGGTCTTGGTG
asmA-F	TGAGACGATTTCTGACGACGCTGATGATTCTCCTGGTCGTGCTGGTGGCCTGTGTAGGCTGGAGCTGCTTCG
asmA-R	GGCCTGCTGCACCAGTTGCTGGAAATTCATTCCTTCAAGGCGCGTATTGCCATATGAATATCCTCCTTAG
wzxC-F	GGCGCTAAATGGTCGGCTATCGCCACGATAGTGATTATCGGTCTGGGGTTTGTGTAGGCTGGAGCTGCTTCG
wzxC-R	CCCCGCCAGATGGCCGCCAATGAGAATTGCCGGGATAAACAGAAACGTTTCATATGAATATCCTCCTTAG
yhjJ-F	GATCGTATTGAAGTTCGTCTCCAGGTTAATACCGGTTCGCTCACCGAAAGTATGTGTAGGCTGGAGCTGCTT CG
yhjJ-R	GATAGCTGCTGACGTAAATTCTGATTGAGCATATCAACGGTCAGGCTGTTGACATATGAATATCCTCCTTAG
yhjK-F	CTGGTACAGCAGAACCGCTACAACACGGCTACGCAACTGGAAAGCATCGCTGTGTAGGCTGGAGCTGCTTCG
yhjK-R	CAGGGTGCATGAGCTGTAACGCGGAAAGATTGACGGAGAGCGGCAATGTCCATATGAATATCCTCCTTAG

Supplementary References

1. Kalonji LM*, et al.* Invasive *Salmonella* Infections at Multiple Surveillance Sites in the Democratic Republic of the Congo, 2011-2014. *Clin Infect Dis* **61 Suppl 4**, S346-353 (2015).

2. Ley B*, et al.* Invasive *Salmonella enterica* serotype typhimurium infections, Democratic Republic of the Congo, 2007-2011. *Emerg Infect Dis* **20**, 701-704 (2014).

3. Lunguya O*, et al.* Antimicrobial resistance in invasive non-typhoid *Salmonella* from the Democratic Republic of the Congo: emergence of decreased fluoroquinolone susceptibility and extended-spectrum beta lactamases. *PLoS Negl Trop Dis* **7**, e2103 (2013).

4. Feasey NA*, et al.* Drug resistance in *Salmonella enterica* ser. Typhimurium bloodstream infection, Malawi. *Emerg Infect Dis* **20**, 1957-1959 (2014).

5. Kariuki S, Onsare RS. Epidemiology and Genomics of Invasive Nontyphoidal *Salmonella* Infections in Kenya. *Clin Infect Dis* **61 Suppl 4**, S317-324 (2015).

6. Msefula CL*, et al.* Genotypic homogeneity of multidrug resistant *S.* Typhimurium infecting distinct adult and childhood susceptibility groups in Blantyre, Malawi. *PLoS One* **7**, e42085 (2012).

7. Leekitcharoenphon P*, et al.* Genomics of an emerging clone of *Salmonella* serovar Typhimurium ST313 from Nigeria and the Democratic Republic of Congo. *J Infect Dev Ctries* **7**, 696-706 (2013).

8. Okoro CK*, et al.* Intracontinental spread of human invasive *Salmonella* Typhimurium pathovariants in sub-Saharan Africa. *Nat Genet* **44**, 1215-1221 (2012).

9. Kingsley RA*, et al.* Epidemic multiple drug resistant *Salmonella* Typhimurium causing invasive disease in sub-Saharan Africa have a distinct genotype. *Genome Res* **19**, 2279-2287 (2009).

10. Kariuki S*, et al.* Ceftriaxone-resistant *Salmonella enterica* serotype typhimurium sequence type 313 from Kenyan patients is associated with the *blaCTX-M-15* gene on a novel IncHI2 plasmid. *Antimicrob Agents Chemother* **59**, 3133-3139 (2015).

11. Romling U. Characterization of the rdar morphotype, a multicellular behaviour in *Enterobacteriaceae*. *Cell Mol Life Sci* **62**, 1234-1246 (2005).

12. Kritikos G*, et al.* A tool named Iris for versatile high-throughput phenotyping in microorganisms. *Nat Microbiol* **2**, 17014 (2017).

13. Gilmour MW, Thomson NR, Sanders M, Parkhill J, Taylor DE. The complete nucleotide sequence of the resistance plasmid R478: defining the backbone

components of incompatibility group H conjugative plasmids through comparative genomics. *Plasmid* **52**, 182-202 (2004).

14. Wheeler NE, Barquist L, Kingsley RA, Gardner PP. A profile-based method for identifying functional divergence of orthologous genes in bacterial genomes. *Bioinformatics* **32**, 3566-3574 (2016).
