# Supplementary material for: An African Salmonella Typhimurium ST313 sublineage with extensive drug-resistance and signatures of host adaptation
Source: Nat Commun. 2019 Sep 19;10:4280. doi: 10.1038/s41467-019-11844-z (PMC6753159; doi:10.1038/s41467-019-11844-z)
Supplement: Supplementary file 2 — Reporting Summary [file 41467_2019_11844_MOESM2_ESM.pdf]

nature research

Corresponding author(s): Sandra Van Puyvelde

Last updated by author(s): Aug 2, 2019

Reporting Summary

Nature Research wishes to improve the reproducibility of the work that we publish. This form provides structure for consistency and transparency in reporting. For further information on Nature Research policies, see Authors & Referees and the Editorial Policy Checklist .

Statistics

For all statistical analyses, confirm that the following items are present in the figure legend, table legend, main text, or Methods section.

n/a	Confirmed	
☐	☒	The exact sample size (n) for each experimental group/condition, given as a discrete number and unit of measurement
☐	☒	A statement on whether measurements were taken from distinct samples or whether the same sample was measured repeatedly
☐	☒	The statistical test(s) used AND whether they are one- or two-sided<br>*Only common tests should be described solely by name; describe more complex techniques in the Methods section.*
☒	☐	A description of all covariates tested
☒	☐	A description of any assumptions or corrections, such as tests of normality and adjustment for multiple comparisons
☒	☐	A full description of the statistical parameters including central tendency (e.g. means) or other basic estimates (e.g. regression coefficient) AND variation (e.g. standard deviation) or associated estimates of uncertainty (e.g. confidence intervals)
☐	☒	For null hypothesis testing, the test statistic (e.g. F, t, r) with confidence intervals, effect sizes, degrees of freedom and P value noted<br>*Give P values as exact values whenever suitable.*
☐	☒	For Bayesian analysis, information on the choice of priors and Markov chain Monte Carlo settings
☒	☐	For hierarchical and complex designs, identification of the appropriate level for tests and full reporting of outcomes
☒	☐	Estimates of effect sizes (e.g. Cohen's d, Pearson's r), indicating how they were calculated

Our web collection on statistics for biologists contains articles on many of the points above.

Software and code

Policy information about availability of computer code

Data collection	Publicly available data:<br>-Salmonella Typhimurium reference genomes of ST313 lineage II (D23580, accession number FN424405.1, (Kingsley et al., 2009))<br>-Illumina sequencing data: accession numbers are listed in supplemental table 2.<br>-Antibiotic resistance database: CARD database version 1.1.8 (McArthur et al., 2013)<br>-gyrA, gyrB, parC, parE, acrB sequences of reference sequence from Salmonella Typhimurium LT2 (NC_003197.2)<br>-plasmid sequences R478 (Serratia marcescens USA, BX664015), pKST313) Salmonella Typhimurium, LN794248) and pSTm-A54650 (Salmonella Typhimurium, LK056646)
Data analysis	Publicly available software:<br>Trimmomatic v.0.33<br>HGAP v3 28 of the SMRT analysis software v2.3.0 (Pacbio, Menlo Park, CA, USA)<br>Circlator v1.1.3 (Hunt et al., 2015)<br>PacBio RS_Resequencing protocol and Quiver v1 of the SMRT analysis software v2.3.0<br>PROKKA v1.11<br>SMALT v0.7.4<br>Picard v1.92<br>samtools mpileup v0.1.19 with parameters “-d 1000 -DSugBf” and bcftools v0.1.19<br>Gubbins v1.4.10<br>snp-sites 2.4.1<br>RAxML v8.2.8<br>Figtree v1.4.2 and iTOL (Letunic and Bork, 2016)<br>BEAST2 v2.4.8<br>Tracer v1.6

LogCombiner v2.4.0
TreeAnnotator v2.4.0
Artemis Comparison Tool (ACT) v16.0.0
BLASTN 2.6.0+
Mauve version 2015_02_25
IRIS software v0.9.7.8
opm package v 1.1.0 in R version 3.3.3
BWA mem v. 0.7.12
ariba v. 2.11.1
SRST2 v. 0.2.0
BRIG (Alikhan et al., 2011)

For manuscripts utilizing custom algorithms or software that are central to the research but not yet described in published literature, software must be made available to editors/reviewers. We strongly encourage code deposition in a community repository (e.g. GitHub). See the Nature Research guidelines for submitting code & software for further information.

Data

Policy information about availability of data

All manuscripts must include a data availability statement. This statement should provide the following information, where applicable:

- Accession codes, unique identifiers, or web links for publicly available datasets
- A list of figures that have associated raw data
- A description of any restrictions on data availability

The sequencing data generated and analysed during the current study are available in the ENA database. The accession codes of the Illumina sequencing data is included in supplemental data 1, the accession code of the Pacbio reference sequence of strain 10433_3 is ERS1310131

Field-specific reporting

Please select the one below that is the best fit for your research. If you are not sure, read the appropriate sections before making your selection.

☐ Life sciences ☐ Behavioural & social sciences ☒ Ecological, evolutionary & environmental sciences

For a reference copy of the document with all sections, see nature.com/documents/nr-reporting-summary-flat.pdf

Ecological, evolutionary & environmental sciences study design

All studies must disclose on these points even when the disclosure is negative.

Study description	Descriptive study of Salmonella Typhimurium isolates obtained from bloodstream infections in the Democratic Republic of the Congo, with quantitative experimental assays on a set of samples.
Research sample	Salmonella enterica subspecies enterica serovar Typhimurium isolates originating from bloodstream infections in the Democratic Republic of the Congo.
Sampling strategy	Out of an original collection of clinical isolates from ongoing surveillance work in the Democratic Republic of the Congo, isolates were selected for molecular and functional analysis based on representative phenotypic characteristics. Minimal numbers for surveillance of AMR were those listed by the clinical laboratory standards document (M39-A4).
Data collection	Blood cultures were collected and processed in the capital, Kinshasa, and the Bas-Congo province (referral hospital of Kisantu) and later extended to sentinel hospitals in the Oriental Province (University Hospital Kisangani with affiliated hospitals and health centers). For more details, see Kalonji and Post et al., 2015 (CID 2015:61 (Suppl 4)) and Tack et al., 2019, in press (CID). Duplicates from all isolates are regularly shipped to the Institute of Tropical Medicine (ITM) in Antwerp (Belgium), and this duplicate collection is subjected to routine phenotyping (determination of species, serovar and antibiotic susceptibility profile). Based on this information, a representative selection was made for further functional analysis.<br>- All isolates of this selection have been whole-genome sequenced using Illumina HiSeq at the University of Antwerp (Belgium) or the Wellcome Sanger Institute (Cambridge, UK).<br>- One representative isolate has been whole-genome sequenced using PacBio sequencing at the Wellcome Sanger Institute, Cambridge, UK.<br>- All isolates of the selection have been subjected to a qualitative biofilm analysis at the Institute of Tropical Medicine, Antwerp (Belgium).<br>- Four representative isolates have been subjected to a metabolic analysis using the Biolog Phenotype Microarrays at KU Leuven (Belgium).<br>- Ten representative isolates have been subjected to macrophage and mice infections at the Wellcome Sanger Institute (Cambridge, UK).<br>- Four mutant strains have been constructed at the Wellcome Sanger Institute (Cambridge, UK).
Timing and spatial scale	Salmonella isolates were collected between 2007 and 2016 as described in Lunguya et al. 2013 (Plos NTD 7: e2103), Kalonji and Post et al., 2015 (CID 2015:61 (Suppl 4)) and Tack et al., 2019, in press (CID).

Data exclusions	NA
Reproducibility	Biolog experiments were performed with 4 isolates from the 2 groups that were compared. Each isolate was analysed in three independent biological replicates.<br>Mice infections were performed with 5 isolates from the 2 groups that were compared, with 5 mice per group. The infections were performed twice.
Randomization	NA
Blinding	NA

Did the study involve field work? ☒ Yes ☐ No

Field work, collection and transport

Field conditions	Low-income country, tropical climate, rainy and dry season alternating and dependent on location above and below the Equator. For reference, see Kalonji and Post 2015 (CID 2015:61 (Suppl 4)), Figure 3. Relevant country indices of the Democratic Republic of the Congo are the median age of the population: 17 years, the human development index: 0:435 (world rank 176/188), the percentage of the population living below income poverty line of $1.9/day: 77.1% (The World Factbook. Congo, Democratic republic of the 2018: https://www.cia.gov/library/publications/the-world-factbook/geos/cg.html; World Health Organization. Democratic Republic of the Congo: WHO statistical profile. 2015: http://www.who.int/countries/cod/en/; The World Bank. Data: Congo, Dem. Rep. 2017: http://www.worldbank.org/en/country/drc)
Location	Blood cultures were collected and processed in the capital, Kinshasa, and the Bas-Congo province (referral hospital of Kisantu) and later extended to sentinel hospitals in the Oriental Province (University Hospital Kisangani with affiliated hospitals and health centers). For more details, see Kalonji and Post et al., 2015 (CID 2015:61 (Suppl 4)) and Tack et al., 2019, in press (CID).
Access and import/export	Ethical approval for the microbiological surveillance study was granted by the Institutional Review Board of ITM, the Ethics Committee of Antwerp University, and the Ministry of Health of the DRC. Annual reports were sent to the ethical committees.<br>Isolates are shared via Material Transfer Agreements with INRB and shipments were performed according to IATA norms.<br>Surveillance was integrated in clinical care referral hospitals
Disturbance	Occasional (short) stock ruptures (blood culture bottles) as well as canceling of mutual visits due to security issues

Reporting for specific materials, systems and methods

We require information from authors about some types of materials, experimental systems and methods used in many studies. Here, indicate whether each material, system or method listed is relevant to your study. If you are not sure if a list item applies to your research, read the appropriate section before selecting a response.

Materials & experimental systems

n/a	Involved in the study	
☒	☐	Antibodies
☐	☒	Eukaryotic cell lines
☒	☐	Palaeontology
☐	☒	Animals and other organisms
☐	☒	Human research participants
☒	☐	Clinical data

Methods

n/a	Involved in the study	
☒	☐	ChIP-seq
☒	☐	Flow cytometry
☒	☐	MRI-based neuroimaging

Eukaryotic cell lines

Policy information about cell lines

Cell line source(s)	Cells used for the gentamicin killing assays were THP-1 monocytes from European Collection of Authenticated Cell Cultures, cat no. 88081201
Authentication	n/a
Mycoplasma contamination	n/a
Commonly misidentified lines<br>(See ICLAC register)	n/a

Animals and other organisms

Policy information about studies involving animals; ARRIVE guidelines recommended for reporting animal research

Laboratory animals	C57Bl6n mice were used in the studies. They were female and between the age of 6-8 week.
Wild animals	n/a
Field-collected samples	n/a
Ethics oversight	All project licences go through ethical review by the institutes Animal Welfare and Ethical Review Body AWERB committee before being approved by the UK Home Office. Work is reviewed every 2 years during the the life of the licence.

Note that full information on the approval of the study protocol must also be provided in the manuscript.

Human research participants

Policy information about studies involving human research participants

Population characteristics	Study population in whom Salmonella were obtained from blood cultures comprised approximately 37500 individuals suspected of bloodstream infection. Depending on the year of sampling and study site, pathogens were identified in between 10.0% and 15.0% of the samples, of which 50% were Salmonella. Age and Sex distribution are described in Kalonji and Post 2015 (CID 2015:61 (Suppl 4)). Approximately 50 % of the Salmonella Typhimurium isolates originate from patients < 2 years, with a male-to-female ratio of approximately 1.2. For indications of blood culture sampling see Kalonji and Post 2015 (CID 2015:61 (Suppl 4)).
Recruitment	Patients were recruited based on clinical indications for blood culture sampling. Blood cultures were free-of-charge and integrated in routine patient care (complying with international standards of care). Small variations may have occurred depending on the clinician's interpretation of the indications, but quality indicators of blood cultures were constant over time and sampling site.
Ethics oversight	The study protocol was approved by the Institutional Review Board at the ITM in Antwerp, by the Ethics Committees of the Antwerp University (Belgium) and the School of Public Health (Kinshasa, DRC).

Note that full information on the approval of the study protocol must also be provided in the manuscript.
