# Supplementary material for: An African Salmonella Typhimurium ST313 sublineage with extensive drug-resistance and signatures of host adaptation
Source: Nat Commun. 2019 Sep 19;10:4280. doi: 10.1038/s41467-019-11844-z (PMC6753159; doi:10.1038/s41467-019-11844-z)
Supplement: Supplementary file 3 — Description of Additional Supplementary Files [file 41467_2019_11844_MOESM3_ESM.pdf]

Description of Additional Supplementary Files

File Name: Supplementary Data 1
Description: Metadata of 81 *Salmonella* Typhimurium isolates from the Democratic Republic of the Congo, sequenced in this study. For each isolate, basic clinical information (province, hospital, age group of the patient and year of isolation), sequencing accession IDs, antibiotic susceptibility testing with interpretation according to the CLSI guidelines, antibiotic resistance summary and identified genetic markers are given. MDR = multidrug resistance, DCS = decreased ciprofloxacin susceptibility, ESBL = extended spectrum beta-lactamase, AZI-R = azithromycin resistant.

File Name: Supplementary Data 2
Description: Metadata of all 276 *Salmonella* Typhimurium genomes included in the genomics analysis. For each isolate, basic clinical information (continent, country, year of isolation, source and tissue), sequencing accession ID and study reference, results of genomic MLST typing and annotation of ST313 lineages, results of identified antibiotic resistance markers and plasmid replicons are given.

File Name: Supplementary Data 3
Description: Respiration of sublineage II.1 isolates versus lineage II isolates on 192 carbon compounds, using the Biolog Omnilog system. For each compound, the coefficient and p-value are given of a Tukey-type contract analysis between 4 strains per lineage analysed in 3 biological replicates each.
